# Morphometric analysis and taxonomic revision of *Anisopteromalus* Ruschka (Hymenoptera: Chalcidoidea: Pteromalidae) – an integrative approach

**DOI:** 10.1111/syen.12081

**Published:** 2014-06-12

**Authors:** Hannes Baur, Yvonne Kranz-Baltensperger, Astrid Cruaud, Jean-Yves Rasplus, Alexander V Timokhov, Vladimir E Gokhman

**Affiliations:** 1 Abteilung Wirbellose Tiere, Naturhistorisches Museum der Burgergemeinde BernBern, Switzerland; 2 INRA, UMR1062 CBGPMontferrier-sur-Lez, France; 3 Department of Entomology, Moscow State UniversityMoscow, Russia; 4 Botanical Garden, Moscow State UniversityMoscow, Russia

## Abstract

We use an integrative taxonomic approach to revise the genus *Anisopteromalus*. In particular, we apply multivariate ratio analysis (MRA), a rather new statistical method based on principal component analysis (PCA) and linear discriminant analysis (LDA), to numerous body measurements and combine the data with those from our molecular analysis of *Cytb* and *ITS2* genetic markers (on a subset of species) and all available published data on morphology, karyology, behaviour, host associations and geographic distribution. We demonstrate that the analysis of quantitative characters using MRA plays a major role for the integration of name-bearing types and thus for the association of taxa with names. Six species are recognized, of which two are new: *A. cornis* Baur **sp.n.** and *A. quinarius* Gokhman & Baur **sp.n.** For *Anisopteromalus calandrae* (Howard), a well-known, cosmopolitan parasitoid of stored-product pests, we have selected a neotype to foster continuity and stability in the application of this important name. The species was sometimes confused with the related *A. quinarius* **sp.n.**, another cosmopolitan species that is frequently encountered in similar environments. We also show that several species originally described or later put under *Anisopteromalus* actually belong to different genera: *Cyrtoptyx camerunus* (Risbec) **comb.n.**; *Meraporus glaber* (Szelényi) **comb.n.**; *Dinarmus schwenkei* (Roomi, Khan & Khan) **comb.n.** *Neocatolaccus indicus* Ayyar & Mani is confirmed as a junior synonym of *Oxysychus sphenopterae* (Ferrière) **syn.n.** and *Anisopteromalus calandrae brasiliensis* (Domenichini) **stat.rev.** must be considered as a valid but doubtful taxon.

This published work has been registered in ZooBank, http://zoobank.org/urn:lsid:zoobank.org:pub:BDFE96D3-D0F4-4012-90F5-9A087F7F5864.

## Introduction

In systematics, data from multiple sources are becoming more and more easily available to taxonomists working at the species level. This has led to interest in a method for combining these data, which recently has been termed integrative taxonomy (for a review and the distinction from iterative taxonomy, see Yeates *et al.*, 2011). The term is adopted when species delimitation is based on results from a variety of disciplines, for instance morphology, DNA analysis, cytogenetics, behaviour or biochemistry. Detailed procedures have been devised for integrating conflicting datasets into sound hypotheses (e.g. Schlick-Steiner *et al.*, 2010).

In most studies adopting an integrative approach, attention is usually paid just to species limits *per se*, based on the evaluation of the sample at hand (e.g. Chesters *et al.*, 2012). Only rarely is the main task of a taxonomic revision also considered (e.g. Steiner *et al.*, 2010), that is, the association of taxa with names. This leads to cases where information is often very limited and data integration becomes a real challenge: for example, when name-bearing types are damaged and character sets are thus incomplete; when types are too fragile or old for molecular analyses; and when types are lost (or inaccessible) and nominal taxa are based on nothing more than a set of qualitative characters. In combination such challenges cause major, and widespread, problems in evaluating available evidence to support necessary taxonomic revisions.

Although focusing on morphometry, we are here pursuing such an integrative approach for establishing species boundaries and identities within *Anisopteromalus* Ruschka (Hymenoptera: Chalcidoidea: Pteromalidae: Pteromalinae), a small genus of parasitic wasps currently comprising seven species (Noyes, 2013). The genus was erected for *Anisopteromalus mollis* Ruschka which is the type species by monotypy, a species synonymized with *A. calandrae* (Howard) by Graham (1969). The genus is easily recognized by a combination of characters, most notably the female antenna with three anelli, the structure of the propodeum and the extended hind margin of the first gastral tergite (Graham, 1969; Bouček & Rasplus, 1991). Species of *Anisopteromalus* occur mainly in the Old World, where they were recorded from tropical Africa (Risbec, 1956; Rasplus, 1988), Asia (Roomi *et al.*, 1973; Sureshan, 2010) and Western Europe (Szelényi, 1981). They usually parasitize beetle larvae (e.g. Chrysomelidae: Bruchinae, Anobiidae, Curculionoidea) feeding on stored grain and legume seeds (Fabaceae: Faboideae and Caesalpinioideae), but have sometimes been reared also from lepidopteran hosts (e.g. Gelechiidae, Pyralidae) (Noyes, 2013).

While for most *Anisopteromalus* species hardly anything has been published beside the original description or an occasional host record, *A. calandrae* is a well-known, cosmopolitan parasitoid of various stored-product pests. It has been the subject of numerous studies spanning a wide variety of topics, such as biological control (Hou *et al.*, 2004; Ngamo *et al.*, 2007; Ni *et al.*, 2008; Chaisaeng *et al.*, 2010), impact of pesticides and herbicides (Perez-Mendoza *et al.*, 1999; Lacoume *et al.*, 2009; Yoon *et al.*, 2009), life-history traits (Bressac *et al.*, 2009; Lebreton *et al.*, 2009a, 2010; Chaisaeng *et al.*, 2010), behaviour including learning (Ryoo *et al.*, 1996; Lebreton *et al.*, 2009b; Belda & Riudavets, 2010; Ishii & Shimada, 2010) and physiology (Zhu *et al.*, 1999; Howard & Baker, 2003). The ISI Web of Science database (Thomson Reuters) cites more than 100 papers in this respect and many of the older works can be retrieved from the Universal Chalcidoidea Database (Noyes, 2013).

Despite the multitude of studies, little critical attention has been paid to the systematics of *Anisopteromalus*. As mentioned before, most species have not been re-considered since their description. Furthermore, the taxonomic status of *A. calandrae* is doubtful. Gokhman *et al.* (1998) first suspected that two sibling species might be hidden under that name. In fact, investigation of the karyotypes of two laboratory strains revealed a marked difference in the number and shape of chromosomes. One strain originating from Moscow, Russia (called the MSU strain) showed five chromosomes in its haploid set (*n* = 5). All of those chromosomes were metacentric except for the last submetacentric one. Moreover, they could be subdivided into two size classes. In the other strain, cultured at Imperial College at Silwood Park, Ascot, Berkshire, UK (origin Slough, Berkshire; called the ICSP strain) with *n* = 7, all chromosomes were metacentric and demonstrated a continuous gradation in length. Such differences in the karyotype are usually a clear indication of different species (Gokhman, 2009). Gokhman *et al.* (1998, 1999) also found significant differences in morphology, courtship behaviour and several important life-history traits. Because the crossing between MSU and ICSP strains also failed, there could be little doubt that more than one taxon was involved. Later on, similar differences between some other populations presumably belonging to *A. calandrae* were also found (Gokhman & Timokhov, 2002) and their different host preferences were revealed also (Timokhov & Gokhman, 2003). However, the presence of the two closely related and widely distributed species with contrasting life-history strategies in the genus *Anisopteromalus* got relatively little support from the expert community for a number of years (but see Quicke, 2002), although it is becoming increasingly accepted now (see, e.g., Sasakawa *et al.*, 2012, 2013). Of course, taxonomic ambiguity within such an important taxon needs to be solved.

As mentioned above, we focused on morphometric data and their integration with molecular data as well as all relevant published information on morphology, karyology, behaviour, distribution and ecology. We also considered all valid names and their junior synonyms (in total 16 nominal taxa) and checked their name-bearing types whenever possible. Beside the type material, more than one thousand specimens from various collections and from several cultured strains of supposed ‘*A. calandrae*’ were studied. For the morphometric study we applied multivariate ratio analysis (MRA), a recently developed method that allows the interpretation of results from principal component analysis (PCA) and linear discriminant analysis (LDA) in terms of body ratios (Baur & Leuenberger, 2011) and that is thus especially suited for analysing body measurements in a taxonomic context (reviewed in László *et al.*, 2013). Furthermore, the MRA algorithms offer separate analyses of shape and size as well as an estimation of the extent of shape change with size (i.e. allometry in the sense of Gould, 1966). Allometric variation of body ratios was first observed for Pteromalidae by Janzon (1986) who discussed its impact on species delimitation in the *Pteromalus albipennis* group. MRA also allowed the inclusion of the available name-bearing types, which were not usable for DNA sequencing because the age of specimens (usually more than 20, up to 100 years old) is likely to hamper successful extraction and amplification. We also used the mitochondrial cytochrome b (*Cytb*) and the nuclear internal transcribed spacer 2 (*ITS2*) for differentiating entities within the ‘*A. calandrae*’ complex.

## Material and methods

### Specimens and character selection

For the morphological investigation we used a total of more than 1300 specimens. Morphometric analysis was based on a subset of 289 dry-mounted females. We focused on females because they are usually easier to separate and more readily available (e.g. Graham, 1969; Bouček & Rasplus, 1991). Males were nevertheless studied when necessary, for instance to solve the identity of a nominal taxon that was based on the male sex. The examined material originated from: The Natural History Museum, London, UK (BMNH); Centre de Biologie pour la Gestion des Populations, Montferrier-sur-Lez, France (CBGP); Swiss Federal Institute of Technology, Entomology Collection, Zurich, Switzerland (ETHZ); Muséum d'histoire naturelle, Geneva, Switzerland (MHNG); Muséum national d'Histoire naturelle, Paris, France (MNHN); Natural History Museum, Vienna, Austria (NHMV); Naturhistorisches Museum der Burgergemeinde Bern, Berne, Switzerland (NMBE); Queensland Museum, Brisbane, Australia (QMB); Bohart Museum of Entomology, University of California, Davis, California, USA (UCD); United States National Museum, Washington DC, USA (USNM); Zoological Institute of the Russian Academy of Sciences, St. Petersburg, Russia (ZIN); Zoological Museum of Moscow State University, Moscow, Russia (ZMMU).

Gibson (1997) is followed for terminology of morphological structures. The list of morphometric characters used in the analyses is given in Table1. Table S1 gives an overview of the basic descriptive statistics for each measurement (in µm) and species as well as the sample sizes. The selected characters correspond to those used in the taxonomy of Pteromalidae for calculating standard ratios (e.g. Graham, 1969). Most measurements were made with an Olympus SZ11 stereomicroscope at different magnifications using a calibrated eye-piece micrometer (12 mm subdivided into 120 units) and were taken by Y. Kranz-Baltensperger. For some of the name-bearing types, each character was photographed with a Keyence VHX 2000 digital photo-microscope and a VH-Z20R/W zoom lens at a magnification of 200× (i.e. 1000 µm corresponded to 888 pixels) and was measured by H. Baur using ImageJ v1.47v (Schneider *et al.*, 2012); body parts on the images were enlarged 3–4 times before measuring. For all measurements, we ensured that the points of reference were equidistant from the lens of the microscope and that the diaphragm of the lens was fully open. To avoid additional variation due to fluctuating asymmetry (e.g. Palmer & Strobeck, 1986; Bechshøft *et al.*, 2008), measurements of paired characters were usually taken on the left-hand side. The Keyence microscope with a VH-Z100UR/W zoom lens was used for making stack-images of body parts, except for the forewings, which were removed and embedded in Hoyer's medium on slides prior to stack-imaging using a Leica DFC420 camera on a Zeiss Axioskop 40 light microscope and the ImageAccess software (Imagic AG, Glattbrugg, Switzerland).

**Table 1:** Abbreviation, name, definition and magnification of the 20 measurements used for the morphometric analyses of *Anisopteromalus* females.

Abbreviation	Character name	Definition	Magnification
eye.b	Eye breadth	Greatest breadth of eye, lateral view	150×
eye.d	Eye distance	Shortest distance between eyes, dorsal view	150×
eye.h	Eye height	Greatest length of eye height, lateral view	150×
gst.b	Gaster breadth	Greatest breadth of gaster, distance between the outermost lateral edges of the gaster, dorsal view	100×
gst.l	Gaster length	Length of gaster along median line from posterior edge of nucha to tip of ovipositor sheath, dorsal view	70×
hea.b	Head breadth	Greatest breadth of head, dorsal view	100×
hea.h	Head height	Distance between lower edge of clypeus and lower edge of anterior ocellus, frontal view	100×
msc.b	Mesoscutum breadth	Greatest breadth of mesoscutum just in front of level of tegula, dorsal view	100×
msc.l	Mesoscutum length	Length of mesoscutum along median line from posterior edge of pronotum to posterior edge of mesoscutum, dorsal view	150×
msp.l	Malar space	Distance between the point where malar sulcus enters mouth margin and malar sulcus enters lower edge of eye, lateral view (Graham, 1969)	150×
mss.l	Mesosoma length	Length of mesosoma along median line from anterior edge of pronotum collar to posterior edge of nucha, dorsal view	70×
mv.l	Marginal vein	Length of marginal vein, distance between the point at which the submarginal vein touches the leading edge of the wing and the point at which stigmal vein and postmarginal vein unite (Graham, 1969)	150×
ool.l	OOL	Shortest distance between posterior ocellus and eye margin, dorsal view (Graham, 1969)	150×
pdl.flg	Pedicel + flagellum	Combined length of pedicel plus flagellum, outer aspect (Graham, 1969)	100×
pol.l	POL	Shortest distance between posterior ocelli, dorsal view (Graham, 1969)	150×
ppd.l	Propodeum length	Length of propodeum measured along median line from anterior edge to posterior edge of nucha, dorsal view	150×
scp.l	Scape length	Length of scape exclusive of radicle, outer aspect (Graham, 1969)	150×
sct.l	Scutellum length	Length of scutellum along median line from posterior edge of mesoscutum to posterior edge of scutellum, dorsal view	150×
stv.l	Stigmal vein	Length of stigmal vein, distance between the point at which stigmal vein and postmarginal vein unite apically, and the distal end of the stigma (Graham, 1969)	150×
tb3.l	Metatibia	Length of metatibia, measured along midline, outer aspect	100×

### Morphometric analysis

We applied multivariate ratio analysis (MRA) of Baur & Leuenberger (2011) to our data. MRA comprises a set of tools for analysing size and shape of body measurements in a multivariate mathematical framework that is entirely consistent with the customary usage of body lengths and ratios in taxonomic works (e.g. descriptions, diagnoses). In systematic studies, MRA offers several advantages over conventional explorative multivariate methods, such as principal component analysis (PCA) and linear discriminant analysis (LDA). We refer to László *et al.* (2013) who gave an overview of the various MRA tools and applied them to a particular taxonomic problem in some other Hymenoptera. Following Baur & Leuenberger (2011) we first calculated an isometric size axis (isosize), defined as the geometric mean of all variables. We then performed a shape PCA (i.e. a PCA in the space of all ratios) for evaluating how well the morphometric pattern corresponds to the groups obtained by qualitative morphology and karyology. In order to decide how many shape components to retain we inspected the scree graph (Rencher, 2002: 398–399). We also plotted isosize against shape PCs, because the correlation of size with shape is a measure of the amount of allometry in the data (e.g. Klingenberg, 1998). Estimation of the extent of allometric variation is important, because body ratios sometimes correlate with size. Depending on the magnitude of this correlation, the use of ratios may then hold the risk that discrimination of groups is – indirectly – more or less based on size rather than shape (e.g. Janzon, 1986; Seifert, 2008). For this reason, we also employed two graphical tools, the PCA ratio spectrum and allometry ratio spectrum, respectively. Finally, we used the LDA ratio extractor to extract the best ratios for use in the identification key and diagnoses, and calculated the standard distance as well as the measure *δ*.

The R language and environment for statistical computing was used for data analysis (R Core Team, 2013; v3.0.2). In particular, we employed slightly modified versions of the R-scripts provided by Baur & Leuenberger (2011, under ‘Supplementary material’). Pearson product-moment correlation coefficients were calculated with the function ‘cor()’ using the default settings. Scatterplots were generated with the package ‘ggplot2’ (Wickham, 2009). A few specimens, especially some of the name-bearing types, lacked one body part or another. In order to be able to include all specimens in the multivariate analyses, missing values were imputed with the R package ‘mice’, using the default settings of the function ‘mice()’. For the calculation of ratios used in the description and Table2, specimens with missing values were excluded, because imputed values may sometimes produce outliers when calculating ratios.

**Table 2:** First and second-best ratios found by the LDA ratio extractor for separating various groupings of *Anisopteromalus* females.

Group comparison	Best ratios	Range group 1	Range group 2	Standard distance	δ
*apiovorus* – rest	^*^eye.h : sct.l	0.88–1.10	1.05–1.32	9.54	0.18
	^*^hea.b : tb3.l	1.53–1.81	1.15–1.55	8.19	0.20
*calandrae – cornis*	^*^pdl.flg : eye.h	1.77–2.25	2.43–2.69	8.73	0.03
	tb3.l : eye.d	0.96–1.15	1.12–1.19	6.42	0.04
*calandrae – quinarius*	^*^eye.b : ool.l	1.47–2.40	2.33–3.36	8.20	0.16
	mss.l : eye.d	1.49–1.77	1.67–1.98	6.52	0.19
*cala* + *corn – cary* + *ceyl* + *quin*	^*^mss.l : ool.l	5.96–8.14	8.11–11.87	7.25	0.18
	^*^hea.b : eye.d	1.37–1.52	1.48–1.69	5.46	0.22
*ceylonensis – cary* + *quin*	^*^hea.h : eye.b	2.02	2.24–2.74	13.66	0.28
	^*^tb3.l : mv.l	1.56	1.67–2.25	12.05	0.31
*caryedophagus – quinarius*	^*^sct.l : stv.l	1.89–2.52	1.38–2.00	8.05	0.02
	^*^gst.l : ool.l	8.81–12.50	11.38–16.43	6.95	0.02

Ratios marked with ^*^ have very little or no overlap and were thus eligible for use in the identification key and the diagnoses.

### Molecular analysis

#### Taxonomic sampling

We sampled six specimens of *Anisopteromalus calandrae* and three specimens of *A. quinarius* belonging to the main strains used in laboratories (Table S2). Specimens were collected alive and stored in 95% ethanol. Adult specimens were identified to species by V.E. Gokhman, A.V. Timokhov and J.-Y. Rasplus. DNA vouchers are deposited at the CBGP collection, Montferrier-sur-Lez, France. Four outgroup taxa belonging to the pteromalid genera *Nasonia* (three species) and *Pachycrepoideus* (one species) were included to root phylogenetic trees. Cytochrome b *(Cytb)* and *ITS2* sequences from *Nasonia* species were downloaded from GenBank (Table S2).

#### DNA extraction, amplification and sequencing

Total DNA was extracted using standard phenol-chloroform methods (Sambrook *et al.*, 1989). Due to repeated failures of amplification of the Cytochrome c oxidase I Folmer fragment (*COI*, standard barcode) for most specimens*,* we used a long fragment of the Cytochrome b gene (*Cytb*) instead. To investigate potential mitochondrial introgression we also sequenced one nuclear gene, the internal transcribed spacer *ITS2* rRNA.

Genes were amplified as follows:

1. *Cytb:* we used primers CP1 (forward) (5′-GAT GAT GAA ATT GGA TC-3′: Harry *et al.*, 1998), CB1 (forward) (5′-TAT GTA CTA CCA TGA GGA CAA ATA TC-3′: Jermiin & Crozier, 1994) and Tser (reverse) (5′-TAT TTC TTT ATT ATG TTT TCA AAA C-3′: Simon *et al.*, 1994). Using the Promega Taq package, 30 cycles of amplification were performed as follows in 25-µL reaction volumes: denaturation step at 92°C for 1 min, annealing at 48°C for 1 min and 30 s, and extension at 72°C for 1 min.
2. *ITS2*: we used forward primer (5′-TGT GAA CTG CAG GAC ACA TG-3′) and reverse primer (5′-AAT GCT TAA ATT TAG GGG GTA-3′: Campbell *et al.*, 1993). 29 cycles of amplification were performed as follows in 25-µL reaction volumes: denaturation step at 94°C for 1 min, annealing at 50°C for 1 min, and extension at 72°C for 2 min.

PCR products were purified with QIAquick PCR purification kit (Qiagen, Venlo, The Netherlands) and directly sequenced on an ABI 377 automated sequencer using TaqFS and dye-labeled terminators (Perkin–Elmer). CP1 and Tser were used as sequencing primers for *Cytb*. Both strands for each overlapping fragment were assembled using the sequence-editing software Geneious v5.5.7 (Drummond *et al.*, 2012). All sequences were deposited in GenBank (Table S2).

#### Sequence data analyses

All gene regions were aligned with MAFFT 6.864 (Katoh *et al.*, 2005) using the L-INS-i option. *Cytb* alignment was translated to amino acids using MEGA 4 (Tamura *et al.*, 2007) to detect frame-shift mutations and premature stop codons, which may indicate the presence of pseudogenes. Pairwise nucleotide sequence divergences were calculated using a Kimura 2-parameter (K2P) model of substitution (Kimura, 1980) in MEGA 4, using the ‘pairwise-deletion’ of gaps option. The most appropriate model of evolution for each gene region was identified using the Akaike information criterion implemented in MrAIC.pl 1.4.3 (Nylander, 2004). We performed Maximum likelihood (ML) analyses of the two gene regions using MPI-parallelized RAxML 7.2.8. (Stamatakis, 2006a). GTRCAT approximation of models was used for ML bootstrapping (Stamatakis, 2006b) (1000 replicates). Analyses were conducted on a 150-core Linux Cluster at CBGP.

### Data resources

Morphometric raw data files, R-scripts used for calculating the MRA, alignments and photographs of measured characters, as well as of type specimens and their labels, are deposited in the Dryad Data Repository at doi: http://doi.org/10.5061/dryad.km728.

## Results

### Morphometric analysis

For the MRA we focused on six groups that could be separated by qualitative characters (*A. apiovorus*, *A. caryedophagus*, *A. ceylonensis*, *A. cornis*) or by karyotype (*A. calandrae*, *A. quinarius*). At this stage prior to the morphometric and molecular analysis, we deliberately avoided the concept of species and rather interpreted the groups in the sense of operational taxonomic units (OTU). We first performed a series of shape PCAs to see how well the OTUs were supported by variation in shape (Figs 3). A PCA type of analysis is convenient here because it does not require *a priori* assignment of OTUs to particular groups but assumes instead that all OTUs belong to one single group. A PCA thus avoids bias with respect to particular groupings (e.g. Pimentel, 1979; Peters & Baur, 2011). According to the scree graph (not shown), only the first and second shape PC were relevant in all analyses reported below.

**Figure 1:**
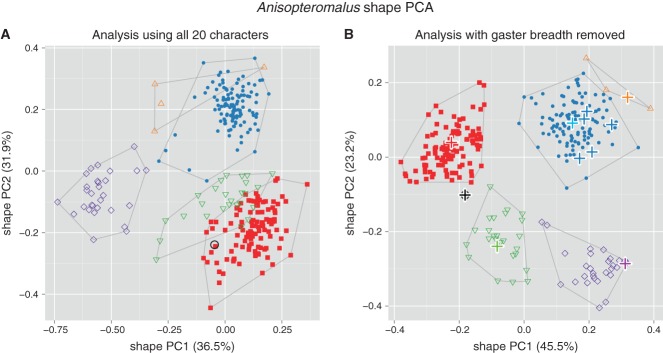
Scatterplot of first against second shape PC of females of all six OTUs of *Anisopteromalus*. (A, B) Shape PCA, including all 20 variables (A) and with variable gaster breadth omitted (B). Closed symbols: blue dots, *A. calandrae*; red squares, *A. quinarius*. Open symbols: violet diamonds, *A. apiovorus*; green upside down triangles, *A. caryedophagus*; black circle, *A. ceylonensis*; orange triangles, *A. cornis*. In (B), name-bearing types are marked with bold plus signs, the light blue plus sign indicates the position of the neotype of *A. calandrae*. The variance explained by each shape PC is given in parentheses.

**Figure 2:**
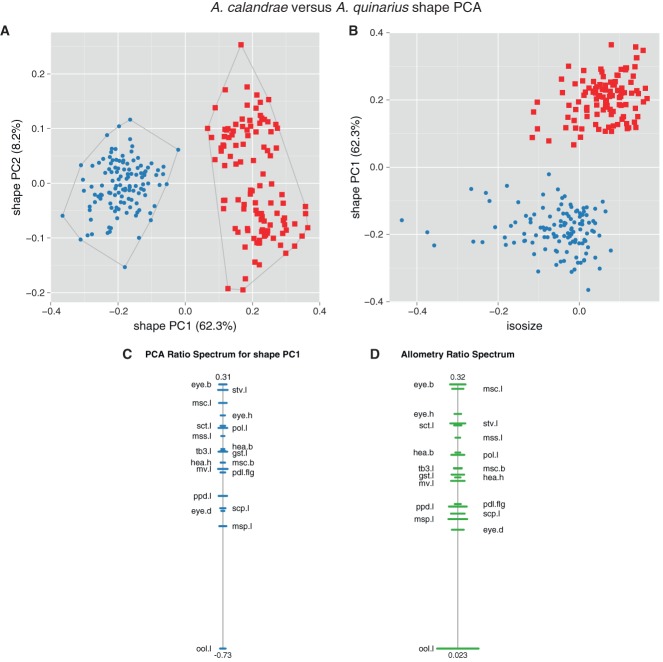
Size and shape analysis of females of OTUs *Anisopteromalus calandrae* and *A. quinarius* using all variables except gaster breadth. (A, B) Shape PCA, scatterplot of first against second shape PC (A), scatterplot of isosize against first shape PC (B). Symbols: blue dots, *A. calandrae*; red squares, *A. quinarius*; in parentheses the variance explained by each shape PC. (C, D) Ratio spectra, PCA ratio spectrum (C), allometry ratio spectrum (D); horizontal bars in the ratio spectra represent 68% bootstrap confidence intervals based on 1000 replicates.

**Figure 3:**
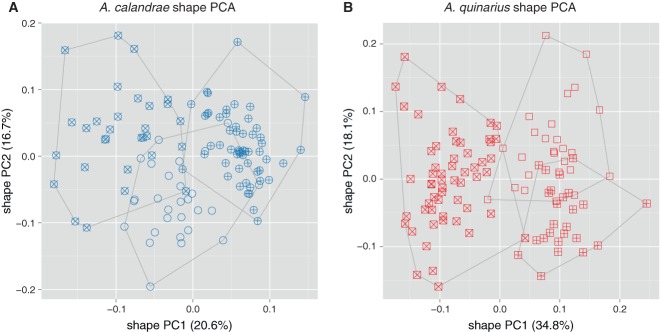
Shape PCA for exploring female variation within *Anisopteromalus calandrae* and *A. quinarius* using all variables except gaster breadth. The analyses included three cultured laboratory strains for each OTU. (A) Scatterplot of first against second shape PC of *A. calandrae*: cross, strain Bamberg USA; plus signs, strain Savannah USA; open circles, strain Slough (ICSP) UK. (B) Scatterplot of first against second shape PC of *A. quinarius*: cross, strain Moscow (MSU) Russia; plus signs, strain Michurinsk Russia; open squares, strain Fresno USA. The variance explained by each shape PC is given in parentheses.

Figure 1A shows a scatterplot of the first two shape PCs including all twenty variables and six OTUs. Only the OTU *A. apiovorus* was clearly distinct while the others were at least slightly overlapping. This result was surprising, as all OTUs could be rather well separated by qualitative morphology. We therefore suspected a significant amount of error variance in some of the variables and checked the variables with matrix scatterplots and Pearson product-moment correlation coefficients. Correlation among body measurements of related taxa should usually be positive and high (e.g. Hills, 1978; Pimentel, 1979), hence its lack may indicate measurement error or morphological artefacts. Indeed, gaster breadth correlated much less strongly than all other variables (Table S3 and Figure S1), with coefficients ranging from −0.18 to 0.30, including many around 0. Closer inspection revealed many specimens with a moderately to strongly deformed gaster (dorso-ventrally or laterally collapsed), mainly in specimens of *A. apiovorus*, *A. caryedophagus*, *A. ceylonensis* and *A. cornis*. The reasons for these artefacts were unclear, as the preservation history of the specimens was unknown. The observed damage nevertheless led us to remove gaster breadth from all further analyses. As a result, in the new graph (Fig. 1B) of a shape PCA with the remaining 19 variables all groups were neatly separated. The plot also shows the position of the available name-bearing types (marked with bold plus signs). Five of them represent some previous synonyms of *A. calandrae* and grouped rather closely with the neotype of *A. calandrae* that we are designating below (see ‘Description of species’ section).

The OTUs *A. apiovorus*, *A. caryedophagus*, *A. ceylonensis* and *A. cornis* were quite distinct based on qualitative morphology alone and were also well supported by morphometric analysis. Therefore, we focused on *A. calandrae* and *A. quinarius* that differ in their karyotypes with the haploid chromosome number of either *n* = 7 or *n* = 5, respectively, but were otherwise generally similar in qualitative morphology. The question thus was how well they were distinguished, not only in shape, but also in size. The results of a shape PCA including only these two OTUs confirmed the pattern in the first plot (Fig. 1B), as they were well separated along the first shape PC (Fig. 2A). In a scatterplot of isosize against the first shape PC (Fig. 2B) *A. quinarius* was on average slightly larger than *A. calandrae*, although the size ranges were broadly overlapping. The plot thus revealed a moderate amount of allometric variation. A similar trend was visible by comparing the PCA ratio spectrum and the allometry ratio spectrum. In a PCA ratio spectrum, only ratios calculated with variables lying at the opposite ends of the spectrum are relevant for a particular shape PC (see also Baur & Leuenberger, 2011; László *et al.*, 2013 for the interpretation of ratio spectra). In the same manner, the most allometric ratios are found in an allometry ratio spectrum. Now, the PCA as well as the allometry ratio spectrum were dominated by the same ratio, eye.b : ool.l (Fig. 2C, D), that is, the most important ratio concerning the first shape PC was also the most allometric one.

The LDA ratio extractor is a tool for finding the best ratios for separating some groups (see Baur & Leuenberger, 2011: 816–818 for how this algorithm works). In contrast to a PCA, group membership had to be specified beforehand. The results are compiled in Table2 showing various comparisons. The ranges of first and second best ratios were often not or only just overlapping between the respective groupings. We were able to integrate such ratios in the identification key and diagnoses (see below), as they represent important diagnostic features.

The best ratio for discrimination of *A. calandrae* from *A. quinarius*, eye.b : ool.l, happened to be the same as the one that dominated the PCA and allometry ratio spectrum (Fig. 2C, D). Note that this is by coincidence, as the best separating ratios must not necessarily correspond to the most important ratios of a PCA ratio spectrum (see also Peters & Baur, 2011 for the conceptual difference between a PCA and a LDA based type of analysis). For instance, the second best ratio, mss.l : eye.d, that still separated most of the specimens in this comparison (compare Table2), was neither in the PCA nor in the allometry ratio spectrum among the dominant ratios (Fig. 2C, D). In fact, the respective variables were lying rather closely to each other in the spectra and thus the ratio had a negligible influence. Now, of the two best discriminating ratios, eye.b : ool.l and mss.l : eye.d, only the former showed allometric behaviour (i.e. correlated with isosize), while the latter did not. This furthermore demonstrates that allometric variation generally had a marginal impact on the discrimination of the OTUs – in other words, separation of *A. calandrae* and *A. quinarius* was attributable to true shape differences, not merely to an indirect size effect.

For all comparisons, δ – a measure of how well shape discriminates in comparison to size (see Baur & Leuenberger, 2011: 818, formula 14) – was close to 0 (0.02–0.31), again indicating that separation of OTUs was mainly due to shape rather than size. Furthermore, standard distances (Baur & Leuenberger, 2011: 817, formula 12) were relatively high, ranging from 5.46–13.66, which reflects the good separation of OTUs observed in the shape PCA (Figs 1B, 2A).

For the exploration of within-group variation we performed a MRA of the three reared strains of *A. calandrae* (Fig. 3A) and *A. quinarius* (Fig. 3B). The scatterplots were in both cases rather homogenous, indicated by the low variation explained by the first two shape PCs (37.3 and 52.9%, respectively). In each OTU, two strains were almost distinct with respect to the first shape PC, but they were partly covered by the third. The analysis did thus not allow any further subdivision of the two OTUs.

### Molecular analysis

*Cytb* (980 bp) and *ITS2* (655 aligned bp) were successfully amplified from all specimens of *A. calandrae* and *A. quinarius*. Alignment of *Cytb* was straightforward due to a lack of length variation, and no stop codons or frame shifts were detected. The intraspecific K2P distance range for *Cytb* was 0.001–0.024 (mean 0.014) for *A. calandrae* and 0.006–0.036 (mean 0.025) for *A. quinarius*. The intraspecific K2P distance range for *ITS2* was 0.000–0.002 (mean 0.001) for *A. calandrae* and 0.002–0.006 (mean 0.004) for *A. quinarius*. Even though these OTUs were morphologically difficult to discriminate, minimum interspecific divergences between *A. calandrae* and *A. quinarius* (*Cytb*: 0.169, *ITS2*: 0.271) were very high, largely exceeding maximum intraspecific divergences. Models chosen by MrAIC were as follows: GTR + I for *Cytb* and SYM + Γ for *ITS2*. As the SYM model is not implemented in RAxML we used GTR instead.

**Figure 4:**
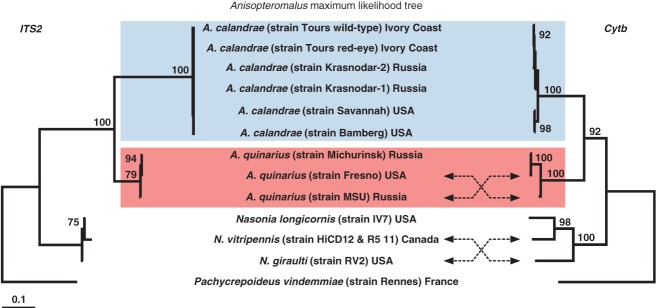
ML trees from the analyses of *ITS2* and *Cytb* sequences of several strains of the OTUs *Anisopteromalus calandrae* and *A. quinarius*. Bootstrap supports higher than 70% are indicated at nodes. Scale bar indicates substitution per site for both trees.

The results of the molecular analysis confirmed the discrimination of *A. calandrae* from *A. quinarius* by MRA. Indeed, phylogenetic analyses of *Cytb* and *ITS2* (Fig. 4) recovered the same well-supported clusters of sequences, which corresponded to both morphologically delineated OTUs (Fig. 2A). Furthermore, comparison between *Cytb* and *ITS2* genetic clusters revealed no mitochondrial introgression between these closely related species.

### Status of OTUs

Our morphometric and molecular analyses unambiguously revealed that all OTUs formed distinct and well-supported taxa. We can thus conclude that the six OTUs examined in this study represent valid species, *A. apiovorus*, *A. calandrae*, *A. caryedophagus*, *A. ceylonensis*, *A. cornis* **sp.n.** and *A. quinarius* **sp.n.** The morphometric analysis furthermore confirmed the synonymy of five nominal taxa with *A. calandrae* (see Fig. 1B). Below, we provide a key to females of all species and descriptions for the two new species as well as for *A. calandrae*. Information on the other species and a discussion of a few doubtful nominal taxa hitherto associated with *Anisopteromalus* can be found in Appendix S1. Nomenclatural changes discussed therein are as follows: *Cyrtoptyx camerunus* (Risbec) **comb.n.**; *Meraporus glaber* (Szelényi) **comb.n.**; *Dinarmus schwenkei* (Roomi, Khan & Khan) **comb.n.**; *Anisopteromalus calandrae brasiliensis* (Domenichini) **stat.rev.**, valid subspecies; *Neocatolaccus indicus* Ayyar & Mani, junior synonym of *Oxysychus sphenopterae* (Ferrière) **syn.n.**

### Key to females

**Figure 5:**
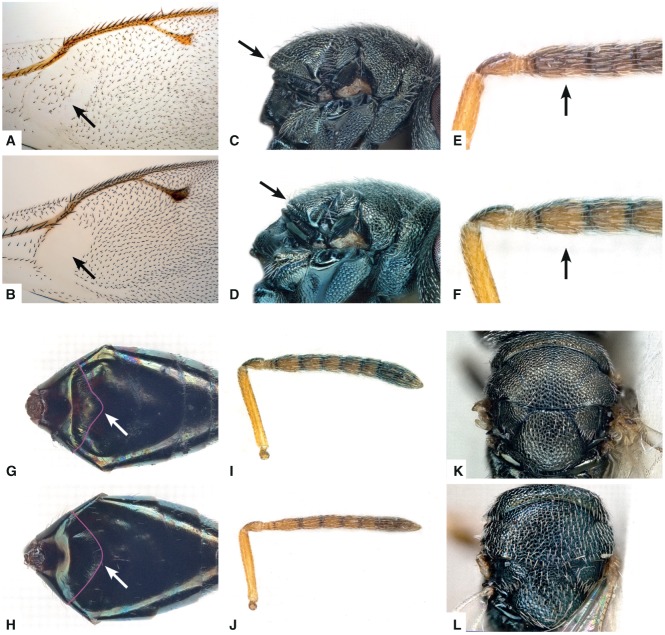
Diagnostic characters of *Anisopteromalus*, females. (A, B) Forewing with speculum, *A. apiovorus* (A; actually the setae are whitish in this species but they appear darkened because of the contrasting background), *A. quinarius* sp.n. (B); (C, D) mesosoma with scutellum projection in lateral view, *A. apiovorus* (C), *A. calandrae* (D); (E, F) proximal funicular segments, *A. apiovorus* (E), *A. calandrae* (F); (G, H) gaster in dorsal view with posterior margin of first tergite emphasized with a magenta line, *A. calandrae* (G), *A. quinarius* sp.n. (H); (I, J) antenna, *A. calandrae* (I), *A. cornis* sp.n. (J); (K, L) mesosoma in dorsal view, *A. calandrae* (K), *A. cornis* sp.n. (L). Arrows mark important character states.

1. Head breadth equal to or more than 1.53× metatibia length **and** eye height equal to or less than 1.1× scutellum length. Forewing speculum medially with a patch of about 10–30 setae (Fig. 5A), setea on wing disc whitish. Scutellum projecting beyond anterior margin of dorsellum (Fig. 5C). First funicular segment subcylindrical, proximally distinctly broader than third anellus and provided with 2–3 rows of longitudinal sensilla (Fig. 5E)…………………………*A. apiovorus* Rasplus

– Either head breadth less than 1.53× metatibia length **or** eye height more than 1.1× scutellum length. Forewing speculum bare (Fig. 5B), setea on wing disc dark. Scutellum projecting at level of anterior margin of dorsellum (Fig. 5D). First funicular segment subconical, proximally at most slightly broader than third anellus and provided with 1–2 rows of longitudinal sensilla (Fig. 5F)…………………………………………2

2. Mesosoma length equal to or less than 8.14× OOL **and** head breadth equal to or less than 1.52× eye distance. Gaster with hind margin of first tergite curving backwards and medially strongly produced (Fig. 5G)…………………………………3

– Mesosoma length more than 8.14× OOL **or** head breadth more than 1.52× eye distance. Gaster with hind margin of first tergite at most slightly curving backwards but medially not produced (Fig. 5H)…………………………………………4

3 . Pedicel plus flagellum 1.77–2.25× eye height; flagellum distinctly clavate (Fig. 5I). Head and mesosoma olive-green, covered with relatively short, inconspicuous whitish setae (Fig. 5K)………………………………*A. calandrae* (Howard)

**Figure 6:**
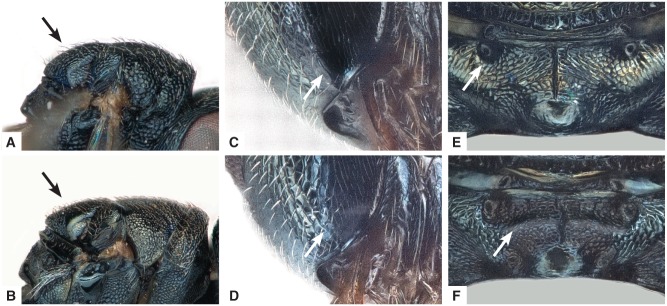
Diagnostic characters of *Anisopteromalus*, females. (A, B) Mesosoma with scutellum shape in lateral view, *A. ceylonensis* (A; holotype female, original position mirrored and photo slightly modified with Photoshop), *A. caryedophagus* (B); (C, D) gena near mouth corner in postero-lateral view, *A. quinarius* sp.n. (C), *A. caryedophagus* (D); (E, F) median area of propodeum, *A. quinarius* sp.n. (E), *A. caryedophagus* (F). Arrows mark important character states.

– Pedicel plus flagellum 2.43–2.69× eye height; flagellum almost filiform (Fig. 5J). Head and mesosoma blue-green, covered with relatively long, conspicuous whitish setae (Fig. 5L)…………………………………*A. cornis* Baur **sp.n.**

4. Scutellum strongly curved in lateral view (Fig. 6A). Head height 2.02× as long as eye breadth. Metatibia only 1.56× as long as marginal vein……………*A. ceylonensis* Sureshan

– Scutellum weakly curved in lateral view (Fig. 6B). Head height distinctly greater, 2.24–2.74× as long as eye breadth. Metatibia longer, 1.67–2.25× as long as marginal vein……5

5. Gena compressed, with a short carina near mouth margin (Fig. 6C). Propodeum with anterior plica consisting of small deep pits, costula indistinct (Fig. 6E). Scutellum length equal to or less than 2.00× stigmal vein **and** gaster length equal to or more than 11.38× OOL*………A. quinarius* Gokhman & Baur **sp.n.**

– Gena terete, not carinate near mouth margin (Fig. 6D). Propodeum with anterior plica short and strongly bent inwards, joining a strong costula (Fig. 6F). Either scutellum length more than 2.00× stigmal vein **or** gaster length less than 11.38× OOL.*A. caryedophagus* Rasplus

**Figure 7:**
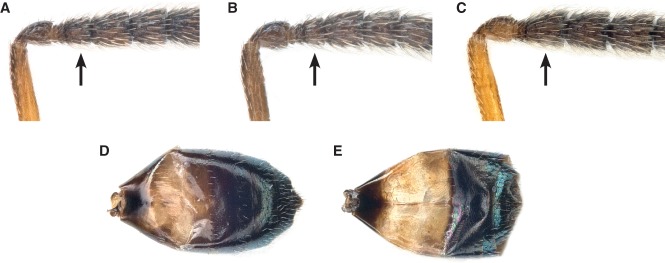
Diagnostic characters of *Anisopteromalus*, males. (A–C) Proximal funicular segments, *A. calandrae*, average-sized specimen (A), the same, small specimen (B), *A. quinarius* sp.n. (C); (D, E) gaster in dorsal view, *A. calandrae* (D), *A. quinarius* sp.n. (E). Arrows mark important character states.

### Description of species (for *A. apiovorus*, *A. caryedophagus* and *A. ceylonensis*, descriptions are provided in Appendix S1)

#### *Anisopteromalus calandrae* (Howard) (Figs 5D, F, G, I, K, 7A, B, D)

1. *Pteromalus calandrae* Howard *in* Comstock, 1881: 273: Neotype ♀ in NMBE, here designated, labelled ‘USA Georgia Savannah Sitophilus oryzae leg. ix. 1978 [print]; chromosomes = 7 strain lab culture e.p. iii.2002 Sitophilus granarius A. Timokhov, Moscow [print]; 1551 Baur [print]; Neotype ♀ Anisopteromalus calandrae Howard lab. H. Baur 2014 [hand, label with red left and right border]’ (entire; glued on a card rectangle); type locality: USA: Georgia, Savannah (examined by Baur). For discussion see under *Remarks*.
2. *Pteromalus oryzae* Cameron, 1891: 184–185: Syntypes ♂♂ and ♀♀ lost (Bouček *et al.*, 1979: 435), but see also Cotes (1896: 11); type locality: INDIA. Synonymized with *A. calandrae* by Bouček *et al.* (1979: 435).
3. Because the original description is inconclusive (Cameron mainly described some colour characters that apply to many *Anisopteromalus* species), we are inclined to accept the synonymy of Bouček *et al.* (1979). Moreover, the species described here as new can be ruled out: first, *A. cornis* **sp.n.** occurs in the Afrotropical region while *P. oryzae* was described from India; second, *A. quinarius* **sp.n.** naturally attacks anobiid beetles, while *P. oryzae* was recorded as a parasite of the rice weevil *Sitophilus oryzae*.
4. *Meraporus vandinei* Tucker, 1910: 343–344: Lectotype ♀ in USNM, catalogue no. 13389, here designated, labelled ‘Plano TX [print] 7. 27. 09 [hand]; emerged on [print] XI. 23. 09 [hand]; Hunter No [print] 1821 [hand]; ESTucker collector [print]; Paratype No. [print] 13389 [hand] U.S.N.M. [print]’ (entire; glued on a card point, with a card rectangle put underneath by Baur); type locality: USA: Texas, Plano (examined by Baur). Synonymized with *A. calandrae* by Mani (1938: 103), synonymy confirmed by MRA (see above).
5. Tucker (1910: 343) stated that his new species was based on 45 specimens collected by himself from Plano and by Van Dine from some other places. He then singled out one female and one male as ‘Type’ and considered the rest to be ‘Paratypes’. However, following the International Code of Zoological Nomenclature (ICZN) all specimens must be considered as syntypes. We received only 6♂ and 33♀ that stood under this name in the USNM. As lectotype we have taken a female with most body parts intact, and not the female with the additional label ‘Meraporus vandinei (n. sp.) Tucker [hand]’ and that emerged on September 11, 1909. This specimen was heavily damaged and lacked the head and wings, but otherwise it was conspecific with the lectotype, as well as all the other paralectotypes we have seen.
6. *Anisopteromalus mollis* Ruschka, 1912: 243–245: Lectotype ♀ in NHMV, here designated, labelled ‘2298 Mehlfruchtbörse [hand]; ex Laemophloeus ferrugineus [hand]; Anisopteromalus mollis m. type. female [hand]; Anisoptero = malus [sic] mollis [hand] det. Ruschka [print]; Type [print, red]’ (entire; lectotype the uppermost specimen on the pin; lectotype and one paralectotype ♀ remounted by Baur on card rectangles); type locality: AUSTRIA: Vienna (examined by Baur). Synonymized with *A. calandrae* by Graham (1969: 435), synonymy confirmed by MRA (see above).
7. *Aplastomorpha pratti* Crawford, 1913: 252–253: Lectotype ♀ in USNM, catalogue no. 15314, here designated, labelled ‘Dallas TX [print], Nov., 1906 [hand]; U.S.D.A. No. [print] 6076 [hand]; Bred from No [print] 3715 [hand]; WDHunter collector [print]; Paratype No. [print] 15314 [hand] U.S.N.M. [print]’ (right antenna beyond third anellus and parts of mid and hind legs lacking; glued on a card point, with a card rectangle put underneath by Baur); type locality: USA: Texas, Dallas (examined by Baur). Synonymized with *A. calandrae* by Mani (1938: 103), synonymy confirmed by MRA (see above).
8. We consider the entire series of 2♂ and 9♀ housed in the USNM to be syntypes, because no specimen was fixed as holotype in the original description (compare Crawford, 1913: 252–253). One female belonged to *Dinarmus*, as also stated by an identification label of Gahan. The rest of the specimens were clearly conspecific. We have taken as lectotype a female with most body parts intact, and not the female with the additional label ‘Aplastomorpha pratti Type Crfd [hand]’. This specimen was in a slightly worse condition, lacking for instance the metatibiae that constitute one of the characters used in our morphometric analyses.
9. *Neocatolaccus australiensis* Girault, 1913: 306: Lectotype ♀ in QMB, catalogue no. 1956, here designated, labelled ‘TYPE [print, orange label]; Syntype 1♂ 2♀♀ Hy. 1956 [hand, red label]; Neocatolaccus australiensis Gir., ♀ types [last word red underlined, Girault hand]’ (head lacking, wings glued to card point and crumpled; glued at tip of a card point; on the same card point two male paralectotypes, one with head missing. Mounted separately on a slide two female and two male heads labelled ‘TYPE [print] Hy/1956 [hand] A. A. Girault [print]; Queensland Museum. [print] Neocatolac ♀ -cus. [sic] australiensis ♂ wing [hand]’; one of the heads might belong to the lectotype, the other one to a paralectotype female mounted on a separate pin; association of those heads with specimens was impossible); type locality: AUSTRALIA: Queensland, Nelson (? = Gordonvale) (examined by Baur). Synonymized with *A. calandrae* by Bouček (1988: 414), synonymy confirmed by MRA (see above). As pointed out by Dahms (1983: 102), Girault (1913) published the catalogue number as ‘1596’ which is wrong. The correct number is ‘1956’ as indicated above.
10. *Bruchobius medius* Masi, 1917: 176–177: Holotype ♀ in BMNH, catalogue no. 5.699, labelled '64 [hand, on card point]; Mahe, '08-9 Seychelles Exp. [print]; Holo- [hand] Type [print; round label with red rim]; Percy Sladen Trust. Exped. B.M. 1913-170. [print]; Brucho = bius medius ♀ Masi [hand]; B.M. TYPE HYM. [print] 5.699; ♀ Anisopteromalus calandrae (How.) [hand] det. Z. Bouček, 197 [print] 5 syn. nov. [hand]' (right flagellum beyond first segment and left hindwing lacking; card point mounted with micro-pin on rectangular tag; head remounted on card point by Baur); type locality: SEYCHELLES: Mahé, Cascade Estate (examined by Baur). Synonymized with *A. calandrae* by Bouček (1976: 22), synonymy confirmed by MRA (see above).
11. *Neocatolaccus mamezophagus* Ishii & Nagasawa, 1942: 67–68: Syntypes ♂♂ and ♀♀ in Tokyo College of Agriculture and Forestry; type locality: JAPAN. Synonymized with *A. calandrae* by Tachikawa (1966: 99).
12. The type material could not be traced, even with the help of Japanese colleagues. The original description clearly rules out that *N. mamezophagus* is the same as any of the new species described here. First, females of *A. cornis* **sp.n.** have the pedicel distinctly shorter than first funicular segment, while it is stated by Ishii & Nagasawa that the pedicel is slightly longer than the first funicular segment; second, males of *A. quinarius* **sp.n.** have the first funicular segment at least as long and usually longer than the second, while the first segment of *N. mamezophagus* is said to be slightly shorter than the second. The character states of *N. mamezophagus* match specimens of *A. calandrae* as defined here, hence we accept the synonymy of Tachikawa (1966).

##### Diagnosis, female

Head and mesosoma olive-green with slight bronze tinges in places, setae whitish, inconspicuous (Fig. 5K). Gena terete, not carinate near mouth margin. Flagellum distinctly clavate (Fig. 5I), first funicular segment subconical, basally slightly broader than third anellus, provided with 1–2 rows of longitudinal sensilla (Fig. 5F). Scutellum projecting at level of anterior margin of dorsellum (Fig. 5D), in lateral view weakly curved. Forewing with setae on wing disc dark. Speculum bare, closed in distal and proximal part. Anterior plica of propodeum short and evenly curved, joining sometimes an indistinct costula. Posterior margin of first gastral tergite curving backwards and strongly produced (Fig. 5G). Head breadth 1.24–1.51× metatibia length and 1.38–1.52× eye distance; head height 2.46–3.39× eye breadth; eye height 1.09–1.30× scutellum length; pedicel plus flagellum 1.77–2.25× eye height; mesosoma length 5.96–8.14× OOL; scutellum length 1.48–1.96× stigmal vein; metatibia length 1.66–2.21× marginal vein; gaster length 7.78–12.02× OOL.

##### Description, female

Antenna with scape testaceous, pedicel testaceous, fuscous on upper side, anelli fuscous and rest of flagellum testaceous, fuscous on upper side. Legs with tibiae yellowish.

Head 1.06–1.19× as broad as mesoscutum. POL 1.22–1.87× OOL. Eyes 1.42–1.80× as high as broad, separated by 1.29–1.59× their height. Malar space 0.46–0.63× eye height. Head in frontal view 1.10–1.30× as broad as high. Antenna with scape 0.77–1.00× as long as eye height. Combined length of pedicel plus flagellum 0.86–1.03× head breadth. First and second anellus strongly transverse, third transverse, about as long as second anellus.

Mesosoma 1.16–1.36× as long as broad. Mesoscutum 1.84–2.42× as broad as long. Hind margin of scutellum broadly rounded. Upper mesepimeron strongly narrowing below, reaching at most basal third of mesopleuron. Basal setal line complete. Costal cell with dorsal surface with at most a short row of setae distally, lower surface with a patch of setae in distal half and a single row of setae running to proximal part, costal setal line complete. Forewing disc moderately pilose. Marginal vein 1.35–1.96× as long as stigmal vein. Stigma subcircular to oval, medium-sized. Propodeum 0.42–0.60× as long as scutellum. Median carina fine, straight. Median area in anterior part evenly reticulate with inner corner of anterior plica with a rather deep and reticulate depression along the anterior plicae. Nucha subglobose, not distinctly separated from rest of propodeum, often at least with some traces of alutaceous sculpture.

Metasoma: Gaster 1.41–2.54× as long as broad, 1.13–1.66× as long as mesosoma, and 0.69–1.22× as long as mesoscutum. Posterior margin of second gastral tergite incised medially to almost straight. Posterior margin of third gastral tergite straight.

##### Material examined

Beside the above-mentioned name-bearing types we examined the following specimens: *Paralectotypes*, *Anisopteromalus mollis*, 2♂, 1♀, same data as lectotype; *Aplastomorpha pratti*, USA: 2♂, 7♀, Texas, Dallas, xi.1906 (*W.D. Hunter*), paratype no. 15314 (USNM); USA: 1♀, Texas, Dallas, xi.1906 (*W.D. Hunter*), type no. 15314 (USNM); *Meraporus vandinei*, USA: 1♂, 1♀, Texas, El Campo, 22.vi.1909 (*D.L. Van Dine*), paratype (USNM); USA: 3♂, 26♀, Texas, Plano, 26.vii.1909 (*E.S. Tucker*), paratype no. 13389 (USNM); USA: 1♂, 1♀, Texas, Plano, 26.vii.1909 (*E.S. Tucker*), type no. 13389 (USNM); USA: 2♀, Louisiana, Welsh, 2.viii.1909 (*D.L. Van Dine*), paratype (USNM); USA: 1♂, 2♀, Louisiana, Lake Arthur, 29.vii.–2.viii.1909 (*Rosenfeld* and *D.L. Van Dine*), paratype (USNM); *Neocatolaccus australiensis*, 2♂, 1♀, same data as lectotype. Further, non-type material is listed in Appendix S1.

##### Biology

The species has a relatively broad host range. It prefers to parasitize *Sitophilus* (Dryophthoridae) and *Callosobruchus* spp. (Chrysomelidae: Bruchinae); however, it can easily be reared (e.g. under laboratory conditions) on certain Anobiidae, like *Lasioderma serricorne* (see Gokhman & Timokhov, 2002; Timokhov & Gokhman, 2003). Many other host records (see Noyes, 2013) need to be verified.

##### Distribution

Cosmopolitan (Noyes, 2013).

##### Remarks

The females of *A. calandrae* can be most easily recognized by the characters given in the key and diagnosis. The species is most similar to *A. cornis* **sp.n.** From *A. quinarius* **sp.n.** it is further distinguished by the pilosity of the forewing, for which we refer to the description of each species.

In order to assess the taxonomic status of *A. calandrae*, it is necessary to concentrate on the other cosmopolitan species, *A. quinarius* **sp.n.** Not only have the two species been confused over a long time, but they both are also likely to occur in human dwellings. However, these species usually occupy somewhat different habitats due to their different host preferences (Gokhman & Timokhov, 2002; Timokhov & Gokhman, 2003): *A. calandrae* inhabits mills and grain bins where it is usually associated with *Sitophilus* spp.; *A. quinarius* **sp.n.** inhabits houses and warehouses (e.g. containing stored fruit or tobacco) being associated with *Stegobium* or *Lasioderma* beetles there. We believe that these differences were an important factor that hampered the discovery of *A. quinarius* **sp.n.** (see Discussion).

A further complication is that the male holotype of *A. calandrae* is now lost (Peck, 1963: 733), which was recently confirmed by the curator of the Chalcidoidea collections at the USNM, M. Gates (personal communication). We have therefore studied characters of the males of the two species that were relevant with respect to Howard's original description. They can be distinguished as follows:

1. *A. calandrae*: First funicular (third flagellar) segment always shorter than the second (Fig. 7A), in small specimens only about as long as combined length of first and second anellus (Fig. 7B). Pale band in proximal half of gaster dirty yellow (Fig. 7D).
2. *A. quinarius* **sp.n.**: First funicular (third flagellar) segment about as long as the second, always much longer than combined length of first and second anellus (Fig. 7C). Pale band in proximal half of gaster bright yellow (Fig. 7E).

Now, Howard *in* Comstock (1881: 273), who apparently examined a very small specimen, noted in his brief but concise description ‘joint 5 [corresponding to the third flagellar segment] small, equal in length to the two ring joints’ and ‘abdomen [corresponding to the gaster] yellow-brown at base’. A comparison of Howard's statements with our description of the male characters should make it clear that both concepts of *A. calandrae* match almost perfectly.

With regard to the overall morphological similarity with the other cosmopolitan species, *A. quinarius* **sp.n.**, we designated a neotype for *A. calandrae* in order to guarantee stability in the application of the name. This is even more important, because *A. calandrae* is the type species of *Aplastomorpha* Crawford, the only synonym of *Anisopteromalus* (see Noyes, 2013). We selected a specimen from Savannah (Georgia), which might appear to be quite far away from Hempstead, Waller County (Texas), the place where the holotype of *A. calandrae* was collected (Howard *in* Comstock, 1881: 273). Given that the species is very easily disseminated from one site to another by transportation of stored products, the distance between the two localities is certainly not a problem here. Taking material of the laboratory culture from Savannah also had the advantage that a specimen in perfect condition coming from the centre of the analysed size and shape space was available (cf. Fig. 1B). The Savannah strain could furthermore be included in our genetic analyses (Fig. 4). Finally, in both cases the specimens were originally reared from *Sitophilus oryzae*-infested crop – that is, both demonstrably had the same host preference.

#### *Anisopteromalus cornis* Baur **sp.n.** (Fig. 5J, L)

http://zoobank.org/urn:lsid:zoobank.org:act:94339C6E-0AF1-4883-836D-BE6AB2EF145B

Holotype ♀ in MHNG, here designated, labelled ‘MALI Sikasso ex stock de mil 1 X 1986 [print, blue label]; B. Sauphanor leg. 6241 [print, blue label]; 2119 Baur [print]; Holotype ♀ Anisopteromalus cornis sp. n. Baur lab. H. Baur 2014 [hand, label with red left and right border]’ (entire; glued on card rectangle with metasoma detached from rest of body); type locality: MALI: Sikasso (examined by Baur).

##### Diagnosis, female

Head and mesosoma blue-green with bronze tinge in places, setae whitish, conspicuous (Fig. 5L). Gena terete, not carinate near mouth margin. Flagellum filiform (Fig. 5J), first funicular segment subconical, basally about as broad as third anellus, provided with 1–2 rows of longitudinal sensilla. Scutellum projecting at level of anterior margin of dorsellum, in lateral view weakly curved. Forewing with setae on wing disc dark. Speculum bare, open below but sometimes closed in proximal part. Anterior plica of propodeum short and evenly curved, joining an indistinct costula. Posterior margin of first gastral tergite curving backwards and strongly produced. Head breadth 1.15–1.26× metatibia length and 1.37–1.40× eye distance; head height 3.15–3.59× eye breadth; eye height 1.05–1.09× scutellum length; pedicel plus flagellum 2.43–2.69× eye height; mesosoma length 6.19–7.96× OOL; scutellum length 1.64–1.74× stigmal vein; metatibia length 1.90–2.29× marginal vein; gaster length 7.98–11.94× OOL.

##### Description, female

Antenna with scape testaceous, pedicel fuscous on upper side, anelli fuscous and rest of flagellum testaceous, fuscous on upper side. Legs with tibiae yellowish.

Head 0.98–1.14× as broad as mesoscutum. POL 1.33–1.64× OOL. Eyes 1.65–1.87× as high as broad, separated by 1.53–1.64× their height. Malar space 0.59–0.74× eye height. Head in frontal view 1.09–1.19× as broad as high. Antenna with scape 1.05–1.10× as long as eye height. Combined length of pedicel plus flagellum 1.06–1.26× head breadth. First and second anellus strongly transverse, third conspicuous, slightly longer than second anellus.

Mesosoma 1.20–1.30× as long as broad. Mesoscutum 1.92–2.16× as broad as long. Hind margin of scutellum broadly rounded. Upper mesepimeron strongly narrowing below, reaching at most basal third of mesopleuron. Basal setal line complete. Costal cell with dorsal surface with a short row of setae distally, lower surface with a patch of setae in distal half and a single row of setae running to proximal part, costal setal line complete. Forewing disc moderately pilose. Marginal vein 1.41–1.78× as long as stigmal vein. Stigma subcircular to slightly elongate, small. Propodeum 0.51–0.57× as long as scutellum. Median carina fine, straight. Median area in anterior part evenly reticulate with inner corner of anterior plica with a rather deep and reticulate depression along the anterior plicae. Nucha subglobose, not distinctly separated from rest of propodeum, alutaceous.

Metasoma: Gaster 1.71–2.89× as long as broad, 1.29–1.50× as long as mesosoma, and 0.63–1.05× as long as mesoscutum. Posterior margin of second gastral tergite weakly incised medially. Posterior margin of third gastral tergite straight.

##### Etymology

The specific name ‘cornis’ is derived from Latin and means ‘horned’. It refers to the relatively long antennae.

##### Material examined

Beside the above-mentioned holotype we examined the following specimens: *Paratypes,* GHANA: 1♂, 1♀, East Gonja District, Salaga, 1944 (*G.S. Cottérell*), ex moth borer on millet seeds (BMNH); MALI: 7♂, 3♀, Sikasso Province, Sikasso, x.1986 (*B. Sauphanor*), ex host on stock millet (CBGP; MHNG; NMBE); NIGERIA: 1♂, 1♀, Kaduna State, Zaria, Samaru, ex *Sitotroga* sp., 20.iii.1958 (BMNH).

##### Biology

Reared from *Sitotroga* sp. (Lepidoptera: Gelechiidae).

##### Distribution

Afrotropical region.

##### Remarks

The species is very close in habitus to *A. calandrae* from which it can be most easily separated by its relatively long flagellum.

#### *Anisopteromalus quinarius* Gokhman & Baur **sp.n.** (Figs 5B, H, 6C, E, 7C, E)

http://zoobank.org/urn:lsid:zoobank.org:act:62905F79-1E91-4D21-88D5-F252B18229F3

Holotype ♀ in NMBE, here designated, labelled ‘RUSSIA Moscow (in Apartment) Stegobium paniceum leg. ix.1995 [print]; chromosomes = 5 strain lab culture e.p. iii.2002 Sitophilus granarius A. Timokhov, Moscow [print]; 1956 Baur [print]; Holotype ♀ Anisopteromalus quinarius sp. n. Gokhman & Baur lab. H. Baur 2014 [hand, label with red left and right border]’ (entire; glued on card point with metasoma detached from rest of body); type locality: RUSSIA: Moscow (examined by Baur).

##### Diagnosis, female

Head and mesosoma olive-green with slight bronze tinge in places, setae whitish, inconspicuous. Gena compressed, with a short carina near mouth margin (Fig. 6C). Flagellum almost filiform, first funicular segment subconical, basally about as broad as third anellus, provided with 1–2 rows of longitudinal sensilla. Scutellum projecting at level of anterior margin of dorsellum, in lateral view weakly curved. Forewing with setae on wing disc dark. Speculum bare, open below but sometimes closed in proximal part (Fig. 5B). Anterior plica of propodeum consisting of small deep pits, costula indistinct (Fig. 6E). Posterior margin of first gastral tergite curving backwards, not produced medially (Fig. 5H). Head breadth 1.24–1.51× metatibia length and 1.48–1.69× eye distance; head height 2.34–2.74× eye breadth; eye height 1.09–1.32× scutellum length; pedicel plus flagellum 1.63–1.97× eye height; mesosoma length 8.11–11.87× OOL; scutellum length 1.38–2.00× stigmal vein; metatibia length 1.67–2.25× marginal vein; gaster length 11.38–16.43× OOL.

##### Description, female

Antenna with scape testaceous, pedicel light brown, first and second anellus testaceous, rest of flagellum fuscous. Legs with tibiae yellowish testaceous or slightly infuscate in basal half.

Head 1.01–1.25× as broad as mesoscutum. POL 1.71–2.46× OOL. Eyes 1.45–1.64× as high as broad, separated by 1.12–1.35× their height. Malar space 0.40–0.53× eye height. Head in frontal view 1.13–1.26× as broad as high. Antenna with scape 0.68–0.85× as long as eye height. Combined length of pedicel plus flagellum 0.84–0.99× head breadth. First and second anellus strongly transverse, third conspicuous, about as long as or longer than first two anelli (shorter in Fresno strain).

Mesosoma 1.18–1.43× as long as broad. Mesoscutum 1.66–2.17× as broad as long. Hind margin of scutellum broadly rounded. Upper mesepimeron strongly narrowing below, reaching at most basal third of mesopleuron. Basal setal line complete. Costal cell with dorsal surface with a patch of setae in distal half and a single row of setae in proximal half, lower surface with a large patch of setae in distal half and two to three rows of setae in proximal part, costal setal line complete. Forewing disc thickly pilose. Marginal vein 1.09–1.90× as long as stigmal vein. Stigma variable in shape, from subcircular (Fresno strain) to subrectangular (MSU strain), large. Propodeum 0.40–0.52× as long as scutellum. Median carina fine, straight. Median area evenly reticulate with inner corner of anterior plica with slight and evenly reticulate depression. Nucha subglobose, not distinctly separated from rest of propodeum, smooth or weakly alutaceous.

Metasoma: Gaster 1.39–2.86× as long as broad, 1.23–1.67× as long as mesosoma, and 0.61–1.21× as long as mesoscutum. Posterior margin of second and third gastral tergite incised medially.

##### Etymology

The specific name ‘quinarius’ is derived from Latin and means ‘of five’. It refers to the haploid chromosome number, which is only five in this species. The name ‘quinarius’ is treated as a noun in apposition and does not change with gender.

##### Material examined

Beside the above-mentioned holotype we examined the following specimens: *Paratypes,* RUSSIA: 3♂, 22♀, Moscow Province, Moscow, ex *Stegobium paniceum* (Linnaeus, 1758), ix.1995, cultured iii.2002 on *Sitophilus granarius* (Linnaeus, 1758), in apartment (NMBE); 22♂, 10♀, Moscow Province, Moscow, ex *Stegobium paniceum* (Linnaeus, 1758), ix.1995, cultured iii.2002 on *Lasioderma serricorne* (Fabricius, 1792), in apartment (NMBE); 6♀, Moscow Province, Moscow, ex *Stegobium paniceum* (Linnaeus, 1758), ix.1995, cultured iii.2002 on *Sitophilus granarius* (Linnaeus, 1758), in apartment (USNM; UCD); 6♂, Moscow Province, Moscow, ex *Stegobium paniceum* (Linnaeus, 1758), ix.1995, cultured iii.2002 on *Lasioderma serricorne* (Fabricius, 1792), in apartment (USNM; UCD); 5♀, Moscow Province, Moscow, ex *Stegobium paniceum* (Linnaeus, 1758), ix.1995, cultured iii.2002 on *Sitophilus granarius* (Linnaeus, 1758), in apartment (BMNH); 1♂, 10♀, Moscow Province, Moscow, ex *Stegobium paniceum* (Linnaeus, 1758), ix.1995, cultured iii.2002 on *Sitophilus granarius* (Linnaeus, 1758), in apartment (ZIN; ZMMU); 12♂, 12♀, Moscow Province, Moscow, ex *Stegobium paniceum* (Linnaeus, 1758), ix.1995, cultured iii.2002 on *Lasioderma serricorne* (Fabricius, 1792), in apartment (ETHZ; MHNG; NHMV); 4♂, 19♀, Tambov Province, Michurinsk, ex *Stegobium paniceum* (Linnaeus, 1758), viii.2000, cultured iii.2002 on *Sitophilus granarius* (Linnaeus, 1758), laboratory culture (NMBE); 2♂, 10♀, Tambov Province, Michurinsk, ex *Stegobium paniceum* (Linnaeus, 1758), viii.2000, cultured iii.2002 on *Sitophilus granarius* (Linnaeus, 1758), laboratory culture (ZIN; ZMMU); USA: 3♂, 24♀, California, Fresno, ex *Lasioderma serricorne* (Fabricius, 1792), 1999, cultured iii.2002 on *Sitophilus granarius* (Linnaeus, 1758), laboratory culture (NMBE); 2♂, 8♀, California, Fresno, ex *Lasioderma serricorne* (Fabricius, 1792), 1999, cultured iii.2002 on *Sitophilus granarius* (Linnaeus, 1758), laboratory culture (ZIN; ZMMU). Further, non-type material is listed in Appendix S1.

##### Biology

Host preferences of this species contrast somewhat with those of *A. calandrae*. Specifically, it prefers to attack certain Anobiidae, for example *Stegobium paniceum* or *Lasioderma serricorne*; however, it can easily be reared (e.g. under laboratory conditions) on *Sitophilus* spp. (Dryophthoridae; see, e.g., Timokhov & Gokhman, 2003).

##### Distribution

Cosmopolitan.

##### Remarks

The females of *A. quinarius* **sp.n.** can be recognized by the characters given in the key and diagnosis. For the separation of males from *A. calandrae*, see ‘Remarks’ in that species description.

## Discussion

### Morphometry and data integration

Multivariate ratio analysis (MRA) revealed six distinct clusters that fully corresponded to the species delineation based on qualitative characters (Fig. 1B). For *A. calandrae* and *A. quinarius* **sp.n.** our DNA analyses as well as the published data on karyotype, behaviour and life-history strategies (summarized in Gokhman & Timokhov, 2002) also confirmed the morphometric differentiation of the two species (see Fig. 2 for interspecific and Fig. 3 for intraspecific variation). Concerning the molecular markers we would like to point to the fact that the comparison between *ITS2* and *Cytb* genetic clusters showed no mitochondrial introgression (Fig. 4). Consequently, *Cytb* should be valuable for identification of the cultured strains of ‘*A. calandrae*’ and accurate assignment of specimens to either *A. calandrae* or *A. quinarius* **sp.n.**

The associations of names with species as delimited here was straightforward for *A. apiovorus*, *A. caryedophagus*, *A. ceylonensis*, *A. cornis* **sp.n.** and *A. quinarius* **sp.n.**, because type specimens were available in good condition and thus could be measured and included in the morphometric analyses. The same was true for other nominal taxa where name-bearing types were available (Fig. 1B). All of these names could be confirmed as synonyms of *A. calandrae*. Cases where the type material was lacking posed rather more difficulties. Here, we took the available evidence from the original description and integrated it with our data. Once again morphometric character ratios proved to be most helpful. For instance measurements taken from drawings of figures published in the original description of *A. calandrae brasiliensis* (Domenichini) allowed us to exclude that this taxon belongs to any of the new species described here (see section on doubtful taxa in Appendix S1). In contrast, where morphometric data were unavailable, the establishment of the identity of a taxon was usually less certain (see, for instance, *Pteromalus oryzae* Cameron, a synonym of *A. calandrae*).

Finally we would like to stress two points with respect to the MRA approach as applied herein. First, its utility in finding the best discriminating ratios for separating various groups could be demonstrated (Table2). Such best ratios functioned as diagnostic characters and formed an essential part of the key and diagnoses. The application of MRA thus allowed a seamless integration of the output from multivariate analysis with the descriptive part of our study. This was also the case in other studies using the MRA technique (László *et al.*, 2013; Huber *et al.*, 2013; Neumeyer *et al.*, 2014; Schweizer *et al.*, 2014; Shirihai *et al.*, 2014) and is clearly advantageous compared to the application of the standard morphometric methods (e.g. Polaszek *et al.*, 2004; Peters & Baur, 2011).

A second point should be mentioned here because we think it often goes unnoticed. It concerns the fact that a single, error-prone variable may have a devastating effect on a morphometric analysis. We demonstrated such an effect in our Fig. 1A where the deformation of the gaster in some specimens led us to remove the variable gaster breadth which resulted in a much improved analysis (Fig. 1B; see László *et al.*, 2013, for why gaster length is much less vulnerable to damage in parasitic wasps). We therefore suggest that the quality of specimens and individual measurements should always be carefully checked by visual inspection, matrix scatterplots and calculation of correlation coefficients (Table S3 and Figure S1).

### Taxonomic implications

The present study obviously demonstrates how little we know about parasitoid biodiversity even in densely populated areas of developed countries, not to mention remote (e.g. tropical) ones. It also raises a question that has general ramifications: why was *A. quinarius* **sp.n.** not discovered earlier? One reason is that because this species is superficially similar to the well-known *A. calandrae*, its presence was not detected until now, although *A. quinarius* **sp.n.** is likely to have also been inhabiting human dwellings for at least a few millennia, as its preferred hosts, including *Stegobium paniceum*, were first recorded there as early as >4000 years ago (see King, 2010). Another reason is that the different host preferences of these two parasitoid species (Timokhov & Gokhman, 2003) led to the situation where quite a few researchers, especially those working in the field of practical pest management, came across *A. calandrae* and *A. quinarius* **sp.n.** simultaneously. Consequently, very few institutions kept both species in culture at the same time, thus preventing the discovery of their reproductive isolation. Furthermore, even in those cases, the observed isolation could be treated as a by-product of continuous adaptation to different host species. Subtle morphological differences between these lineages could also be explained by host influence, both in terms of their genetic background and modification by the environment. A final reason is that the discovery of *A. quinarius* **sp.n.** was hampered by pressure of the taxonomic tradition: all authoritative catalogues and manuals that treat the genus *Anisopteromalus* (see Peck, 1963; Graham, 1969; Bouček, 1988; Bouček & Rasplus, 1991; Noyes, 2013) list *A. calandrae* as its only cosmopolitan species, and strong evidence was therefore needed to call it into question.

Because karyotypic study became a starting point for the present work, we would also like to stress the importance of chromosomal analysis for studying parasitoid taxonomy. Karyotype structure, together with molecular characters, does not depend on environmental conditions, at least not directly. The results obtained, together with a number of similar cases (see Gokhman, 2009 for review), call for wider application of chromosomal analysis to parasitoid stocks cultivated for both industrial and laboratory use. This kind of analysis can provide a means of rapid identification of particular strains, as well as reveal certain karyotypic features that could affect particular genetic and life-history traits.

## Supporting Information

**Figure S1.** Matrix scatterplot of eight variables (in µm) of *Anisopteromalus.*

**Table S1.** Overview of measurements of *Anisopteromalus* females, showing minimum, maximum, mean and standard deviation in µm (except for *A. ceylonensis* with *n* = 1).

**Table S2.** List of *Anisopteromalus* specimens and outgroups included in the molecular analysis.

**Table S3.** Pearson's product-moment correlation coefficients for all measurements of *Anisopteromalus*.

**Appendix S1.** Descriptions of species and lists of further material examined.
