# Supplementary figures and images for: Morphometric analysis and taxonomic revision of Anisopteromalus Ruschka (Hymenoptera: Chalcidoidea: Pteromalidae) – an integrative approach
Source: Syst Entomol. 2014 Jun 12;39(4):691–709. doi: 10.1111/syen.12081 (PMC4459240; doi:10.1111/syen.12081)

Figure S1. Matrix scatterplot of eight variables (in  $\mu\text{m}$ ) of *Anisopteromalus*.

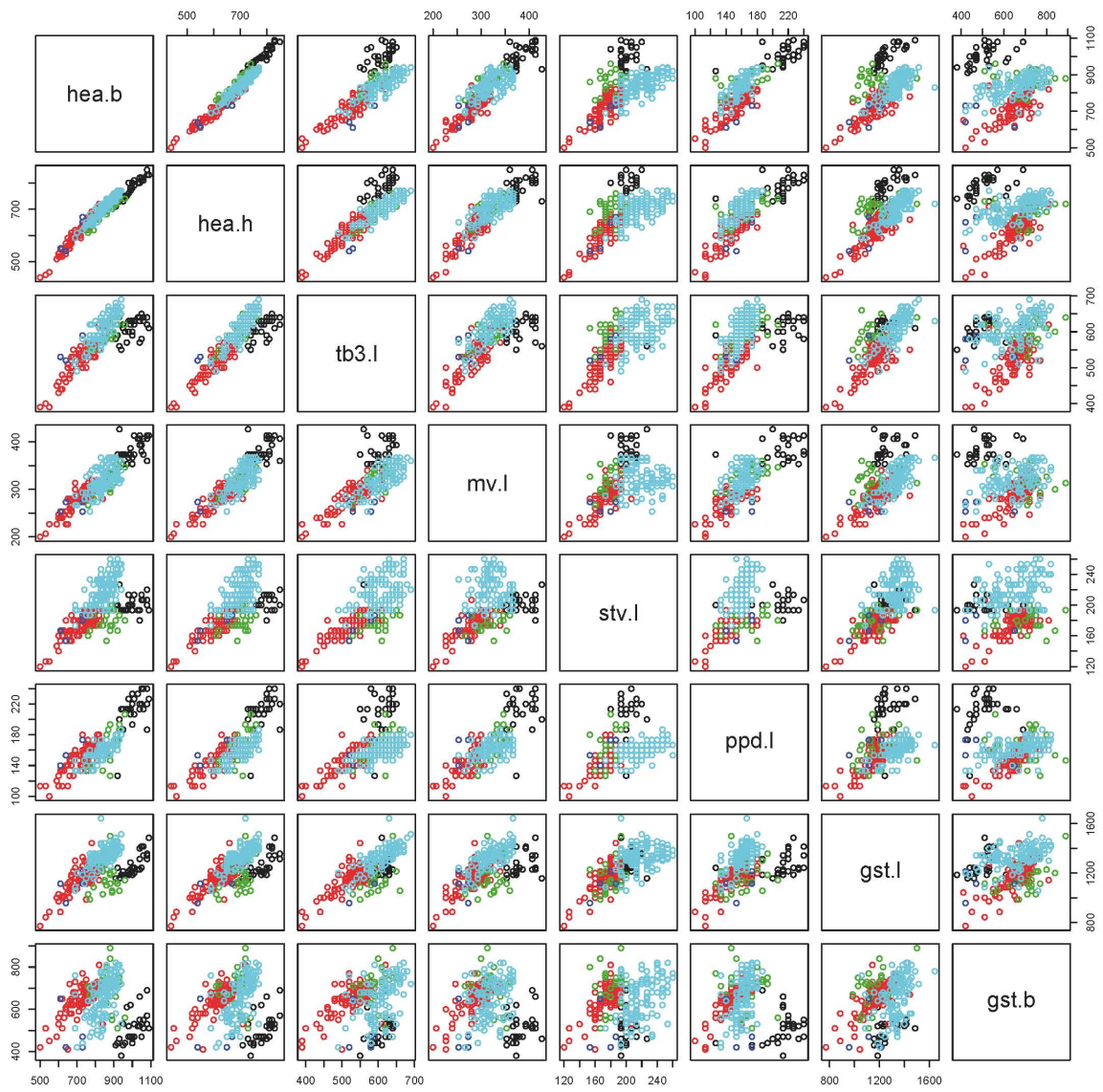

Supplement: Supplementary file 1 — Figure S1. Matrix scatterplot of eight variables (in µm) of Anisopteromalus. [file syen0039-0691-sd1.pdf]
