# Supplementary material for: Morphometric analysis and taxonomic revision of Anisopteromalus Ruschka (Hymenoptera: Chalcidoidea: Pteromalidae) – an integrative approach
Source: Syst Entomol. 2014 Jun 12;39(4):691–709. doi: 10.1111/syen.12081 (PMC4459240; doi:10.1111/syen.12081)
Supplement: Supplementary file 2 — Table S1. Overview of measurements of Anisopteromalus females, showing minimum, maximum, mean and standard deviation in µm (except for A. ceylonensis with n = 1). [file syen0039-0691-sd2.pdf]

Tables and Figures

Table S1. Overview of measurements of *Anisopteromalus* females, showing minimum, maximum, mean, and standard deviation in µm (except for *A. ceylonensis* with n = 1).

	A. apiovorus, n = 28				*A. calandrae*, n = 120			
character	min	max	mean	sd	min	max	mean	sd
eye.b	280	340	314.5	17.03	147	260	222.1	23.82
eye.d	580	707	652.1	36.29	353	580	506.4	42.41
eye.h	413	500	447.9	23.66	227	420	357.3	34.05
gst.b	380	690	504.3	66.58	410	810	650.6	70.30
gst.l	1157	1486	1263.3	89.21	771	1443	1160.3	102.56
hea.b	910	1090	1005.4	52.53	500	840	732.6	65.55
hea.h	710	850	786.4	38.32	440	720	623.0	52.72
msc.b	740	960	861.1	59.59	430	750	649.9	56.93
msc.l	413	527	481.7	27.25	200	400	318.2	33.58
msp.l	220	287	255.0	18.65	127	227	191.3	20.19
mss.l	943	1171	1094.9	57.70	543	957	828.0	73.41
mv.l	353	427	382.4	21.71	200	340	286.8	25.84
ool.l	127	180	160.7	11.67	73	153	119.2	13.11
pdl.flg	740	900	832.5	46.48	510	800	697.1	50.05
pol.l	200	253	225.0	12.78	133	220	180.3	15.75
ppd.l	127	240	211.9	23.47	100	180	151.3	15.39
scp.l	327	393	355.5	20.93	213	360	317.0	28.06
sct.l	400	507	459.3	23.82	200	347	298.0	28.01
stv.l	193	227	202.4	9.29	120	207	175.2	13.72
tb3.l	550	650	612.5	26.75	390	620	531.4	42.88

	A. caryedophagus, n = 26				*A. ceylonensis* n = 1
character	min	max	mean	sd	value
eye.b	247	313	281.3	17.08	233
eye.d	480	580	540.6	27.50	388
eye.h	373	487	439.5	27.74	325
gst.b	510	890	708.4	88.74	417
gst.l	986	1500	1158.2	117.26	1071
hea.b	760	960	867.6	47.38	629
hea.h	610	760	696.5	39.59	472
msc.b	670	890	790.3	55.89	557
msc.l	347	467	422.5	30.79	258
msp.l	187	247	216.5	15.68	140
mss.l	900	1129	1024.1	57.42	666
mv.l	267	360	319.5	22.21	262

ool.l	93	120	109.5	9.06	75
pdl.flg	630	790	720.1	41.59	522
pol.l	187	233	211.8	11.34	158
ppd.l	127	207	163.8	20.35	116
scp.l	293	373	341.0	19.74	219
sct.l	340	427	385.2	18.97	279
stv.l	153	200	180.4	12.32	130
tb3.l	530	660	601.1	31.98	408

	A. cornis sp. n., n = 4				*A. quinarius* sp. n., n = 105			
character	min	max	mean	sd	min	max	mean	sd
eye.b	153	207	183.3	27.49	220	327	282.0	20.94
eye.d	447	520	486.7	38.87	453	587	527.3	28.28
eye.h	280	340	311.7	32.83	340	493	432.4	31.56
gst.b	420	650	490.0	109.24	420	820	657.6	94.40
gst.l	957	1214	1100.0	106.27	1057	1643	1333.1	90.70
hea.b	610	730	677.5	61.85	690	940	842.9	56.27
hea.h	540	670	602.5	67.02	590	770	701.1	41.17
msc.b	570	670	625.0	42.03	560	870	728.1	53.96
msc.l	287	333	311.7	25.17	280	467	392.3	35.87
msp.l	187	220	205.0	14.78	153	227	195.8	14.86
mss.l	743	857	792.9	58.90	757	1100	971.4	67.15
mv.l	253	273	263.3	11.55	253	367	321.3	26.72
ool.l	93	133	118.3	17.53	87	113	100.8	7.26
pdl.flg	680	830	777.5	70.89	610	860	773.0	45.44
pol.l	153	180	168.3	13.74	187	240	215.0	13.49
ppd.l	140	173	160.0	16.33	133	187	160.6	11.23
scp.l	300	360	331.7	32.83	260	380	332.1	24.40
sct.l	267	313	291.7	25.17	273	413	354.2	26.71
stv.l	153	187	171.7	14.78	173	260	221.3	19.06
tb3.l	520	590	555.0	35.12	490	690	609.8	41.53
