# Supplementary material for: Morphometric analysis and taxonomic revision of Anisopteromalus Ruschka (Hymenoptera: Chalcidoidea: Pteromalidae) – an integrative approach
Source: Syst Entomol. 2014 Jun 12;39(4):691–709. doi: 10.1111/syen.12081 (PMC4459240; doi:10.1111/syen.12081)
Supplement: Supplementary file 3 — Table S2. List of Anisopteromalus specimens and outgroups included in the molecular analysis. [file syen0039-0691-sd3.pdf]

Table S2. List of *Anisopteromalus* specimens and outgroups included in the molecular analysis.

| **Voucher number** | **Species** | **Country** | **Strains** | **Hosts** | ***Cytb*** | **ITS2** |
|---|---|---|---|---|---|---|
| JRAS05525_0101 | *A. calandrae* | USA | Bamberg | *Sitophilus oryzae* | KJ027666 | KJ027672 |
| JRAS05528_0101 | *A. calandrae* | Russia | Krasnodar-1 | *Sitophilus granarius* | KJ027668 | KJ027678 |
| JRAS05528_0102 | *A. calandrae* | Russia | Krasnodar-2 | *Sitophilus granarius* | KJ027669 | KJ027679 |
| JRAS05529_0101 | *A. calandrae* | USA | Savannah | *Sitophilus oryzae* | KJ027667 | KJ027677 |
| JRAS05531_0101 | *A. calandrae* | Ivory Coast | Tours red-eye | *Callosobruchus maculatus* | KJ027671 | KJ027681 |
| JRAS05530_0101 | *A. calandrae* | Ivory Coast | Tours wild-type | *Callosobruchus maculatus* | KJ027670 | KJ027680 |
| JRAS05526_0101 | *A. quinarius* | USA | Fresno | *Lasioderma serricorne* | KJ027663 | KJ027673 |
| JRAS05527_0101 | *A. quinarius* | Russia | Michurinsk | *Stegobium paniceum* | KJ027664 | KJ027674 |
| JRAS05524_0101 | *A. quinarius* | Russia | MSU | *Stegobium paniceum* | KJ027665 | KJ027675 |
| - | *N. giraulti* | USA | RV2 | | EU746611 | U02953.1 |
| - | *N. longicornis* | USA | IV7 | | EU746612 | U02957.1 |
| - | *N. vitripennis* | Canada | HiCD12 & R5 11 | | EU746610 | U02960 |
| JRAS05532_0101 | *P. vindemmiae* | France | Rennes | *Delia radicum* | KJ027662 | KJ027676 |
