# Supplementary material for: Morphometric analysis and taxonomic revision of Anisopteromalus Ruschka (Hymenoptera: Chalcidoidea: Pteromalidae) – an integrative approach
Source: Syst Entomol. 2014 Jun 12;39(4):691–709. doi: 10.1111/syen.12081 (PMC4459240; doi:10.1111/syen.12081)
Supplement: Supplementary file 4 — Table S3. Pearson's product-moment correlation coefficients for all measurements of Anisopteromalus. [file syen0039-0691-sd4.pdf]

Table S3. Pearson's product-moment correlation coefficients for all measurements of *Anisopteromalus*.

| character | hea.b | hea.h | pol.l | ool.l | eye.h | eye.b | eye.d | msp.l | scp.l | pdl.flg |
|---|---|---|---|---|---|---|---|---|---|---|
| hea.b | 1.000 | 0.972 | 0.854 | 0.494 | 0.907 | 0.941 | 0.919 | 0.828 | 0.809 | 0.869 |
| hea.h | 0.972 | 1.000 | 0.875 | 0.452 | 0.918 | 0.919 | 0.902 | 0.816 | 0.853 | 0.902 |
| pol.l | 0.854 | 0.875 | 1.000 | 0.171 | 0.886 | 0.881 | 0.710 | 0.621 | 0.713 | 0.768 |
| ool.l | 0.494 | 0.452 | 0.171 | 1.000 | 0.164 | 0.247 | 0.745 | 0.730 | 0.480 | 0.410 |
| eye.h | 0.907 | 0.918 | 0.886 | 0.164 | 1.000 | 0.954 | 0.717 | 0.618 | 0.761 | 0.834 |
| eye.b | 0.941 | 0.919 | 0.881 | 0.247 | 0.954 | 1.000 | 0.767 | 0.656 | 0.714 | 0.830 |
| eye.d | 0.919 | 0.902 | 0.710 | 0.745 | 0.717 | 0.767 | 1.000 | 0.923 | 0.820 | 0.809 |
| msp.l | 0.828 | 0.816 | 0.621 | 0.730 | 0.618 | 0.656 | 0.923 | 1.000 | 0.778 | 0.713 |
| scp.l | 0.809 | 0.853 | 0.713 | 0.480 | 0.761 | 0.714 | 0.820 | 0.778 | 1.000 | 0.809 |
| pdl.flg | 0.869 | 0.902 | 0.768 | 0.410 | 0.834 | 0.830 | 0.809 | 0.713 | 0.809 | 1.000 |
| mss.l | 0.961 | 0.963 | 0.869 | 0.356 | 0.940 | 0.936 | 0.841 | 0.770 | 0.799 | 0.853 |
| msc.l | 0.951 | 0.930 | 0.832 | 0.396 | 0.882 | 0.914 | 0.840 | 0.774 | 0.736 | 0.815 |
| msc.b | 0.958 | 0.941 | 0.809 | 0.485 | 0.881 | 0.889 | 0.889 | 0.832 | 0.809 | 0.828 |
| sct.l | 0.959 | 0.926 | 0.797 | 0.496 | 0.852 | 0.895 | 0.882 | 0.811 | 0.714 | 0.813 |
| tb3.l | 0.825 | 0.875 | 0.866 | 0.146 | 0.905 | 0.856 | 0.679 | 0.615 | 0.784 | 0.827 |
| mv.l | 0.888 | 0.883 | 0.784 | 0.501 | 0.787 | 0.820 | 0.850 | 0.779 | 0.709 | 0.787 |
| stv.l | 0.594 | 0.642 | 0.666 | -0.112 | 0.730 | 0.706 | 0.393 | 0.265 | 0.493 | 0.687 |
| ppd.l | 0.825 | 0.802 | 0.583 | 0.669 | 0.639 | 0.686 | 0.865 | 0.811 | 0.671 | 0.750 |
| gst.l | 0.598 | 0.681 | 0.719 | 0.046 | 0.703 | 0.656 | 0.497 | 0.374 | 0.614 | 0.707 |
| gst.b | 0.028 | 0.104 | 0.125 | -0.182 | 0.220 | 0.073 | -0.017 | -0.027 | 0.234 | 0.083 |

| character | mss.l | msc.l | msc.b | sct.l | tb3.l | mv.l | stv.l | ppd.l | gst.l | gst.b |
|---|---|---|---|---|---|---|---|---|---|---|
| hea.b | 0.961 | 0.951 | 0.958 | 0.959 | 0.825 | 0.888 | 0.594 | 0.825 | 0.598 | 0.028 |
| hea.h | 0.963 | 0.930 | 0.941 | 0.926 | 0.875 | 0.883 | 0.642 | 0.802 | 0.681 | 0.104 |
| pol.l | 0.869 | 0.832 | 0.809 | 0.797 | 0.866 | 0.784 | 0.666 | 0.583 | 0.719 | 0.125 |
| ool.l | 0.356 | 0.396 | 0.485 | 0.496 | 0.146 | 0.501 | -0.112 | 0.669 | 0.046 | -0.182 |
| eye.h | 0.940 | 0.882 | 0.881 | 0.852 | 0.905 | 0.787 | 0.730 | 0.639 | 0.703 | 0.220 |
| eye.b | 0.936 | 0.914 | 0.889 | 0.895 | 0.856 | 0.820 | 0.706 | 0.686 | 0.656 | 0.073 |
| eye.d | 0.841 | 0.840 | 0.889 | 0.882 | 0.679 | 0.850 | 0.393 | 0.865 | 0.497 | -0.017 |
| msp.l | 0.770 | 0.774 | 0.832 | 0.811 | 0.615 | 0.779 | 0.265 | 0.811 | 0.374 | -0.027 |
| scp.l | 0.799 | 0.736 | 0.809 | 0.714 | 0.784 | 0.709 | 0.493 | 0.671 | 0.614 | 0.234 |
| pdl.flg | 0.853 | 0.815 | 0.828 | 0.813 | 0.827 | 0.787 | 0.687 | 0.750 | 0.707 | 0.083 |
| mss.l | 1.000 | 0.967 | 0.952 | 0.948 | 0.876 | 0.844 | 0.651 | 0.773 | 0.637 | 0.141 |
| msc.l | 0.967 | 1.000 | 0.937 | 0.950 | 0.803 | 0.823 | 0.613 | 0.770 | 0.547 | 0.029 |
| msc.b | 0.952 | 0.937 | 1.000 | 0.942 | 0.809 | 0.844 | 0.530 | 0.814 | 0.543 | 0.096 |
| sct.l | 0.948 | 0.950 | 0.942 | 1.000 | 0.767 | 0.864 | 0.528 | 0.835 | 0.498 | -0.028 |
| tb3.l | 0.876 | 0.803 | 0.809 | 0.767 | 1.000 | 0.761 | 0.714 | 0.581 | 0.771 | 0.249 |
| mv.l | 0.844 | 0.823 | 0.844 | 0.864 | 0.761 | 1.000 | 0.450 | 0.766 | 0.585 | -0.011 |
| stv.l | 0.651 | 0.613 | 0.530 | 0.528 | 0.714 | 0.450 | 1.000 | 0.365 | 0.704 | 0.177 |
| ppd.l | 0.773 | 0.770 | 0.814 | 0.835 | 0.581 | 0.766 | 0.365 | 1.000 | 0.420 | -0.097 |
| gst.l | 0.637 | 0.547 | 0.543 | 0.498 | 0.771 | 0.585 | 0.704 | 0.420 | 1.000 | 0.295 |
| gst.b | 0.141 | 0.029 | 0.096 | -0.028 | 0.249 | -0.011 | 0.177 | -0.097 | 0.295 | 1.000 |
