# Supplementary material for: Morphometric analysis and taxonomic revision of Anisopteromalus Ruschka (Hymenoptera: Chalcidoidea: Pteromalidae) – an integrative approach
Source: Syst Entomol. 2014 Jun 12;39(4):691–709. doi: 10.1111/syen.12081 (PMC4459240; doi:10.1111/syen.12081)
Supplement: Supplementary file 5 — Appendix S1. Descriptions of species and lists of further material examined. [file syen0039-0691-sd5.pdf]

SYSTEMATIC ENTOMOLOGY

ELECTRONIC SUPPORTING INFORMATION FOR THE ARTICLE

Morphometric analysis and taxonomic revision of *Anisopteromalus* Ruschka (Hymenoptera: Chalcidoidea: Pteromalidae) – an integrative approach

Hannes Baur[1], Yvonne Kranz-Baltensperger[1], Astrid Cruaud[2], Jean-Yves Rasplus[2], Alexander V. Timokhov[3], Vladimir E. Gokhman[4]

[1] Naturhistorisches Museum der Burgergemeinde Bern, Abteilung Wirbellose Tiere, Bernastrasse 15, CH-3005 Bern, Switzerland, hannes.baur@nmbe.ch.
[2] INRA, UMR1062 CBGP, F-34988 Montferrier-sur-Lez, France.
[3]Department of Entomology and [4]Botanical Garden, Moscow State University, Moscow 119234, Russia.

urn:lsid:zoobank.org:pub:BDFE96D3-D0F4-4012-90F5-9A087F7F5864

Appendix S1

Descriptions of species and lists of further material examined

Anisopteromalus apiovorus Rasplus (Figs 5A, C, E)

Anisopteromalus apiovorus Rasplus, 1988: 120–122: Holotype ♀ in MNHN, labeled "COTE D'IVOIRE Lamto Station 5'02W – 6°13'N 5.XII. **–>** [bold arrow] 9.XII.1985 [print, blue label]; ex *Piezotrachelus* sp. dans les gousses de *Vigna multinervis* Hutch. & Dalz. - n°11 [print, blue label]; *Anisopteromalus apiovorus* n.sp. HOLOTYPE JY. Rasplus det. 88 [print, red label]" (entire; glued on card rectangle); type locality: IVORY COAST: Lamto (examined by Baur).

Diagnosis, female. Head and mesosoma olive-green with slight bronze tinge in places, setae whitish, inconspicuous. Gena terete, not carinate near mouth margin. Flagellum almost filiform, first funicular segment subcylindrical, basally distinctly broader than third anellus, provided with 2–3 rows of longitudinal sensilla (Fig. 5E). Scutellum projecting beyond anterior margin of dorsellum (Fig. 5C), in lateral view weakly curved. Fore wing with setae on wing disc whitish (in Fig. 5A they actually appear darkened but this is an artifact because of the contrasting pale background). Speculum medially with a patch of about 10–30 setae, but widely open below (Fig. 5A). Anterior plica of propodeum short and strongly bent inwards, joining an often distinct costula. Posterior margin of first gastral tergite strongly curving backwards, not produced medially. Head breadth 1.53–1.81 times metatibia length and 1.50–1.59 times eye distance; head height 2.41–2.59 times eye breadth; eye height 0.88–1.10 times scutellum length; pedicel plus flagellum 1.72–1.96 times eye height; mesosoma length 6.34–7.44 times OOL; scutellum length 1.91–2.52 times stigmal vein; metatibia length 1.31–1.78 times marginal vein; gaster length 7.29–9.14 times OOL.

Redescription, female. Antenna with scape testaceous, pedicel testaceous, fuscous on upper side, flagellum testaceous, fuscous on upper side. Legs with protibia yellowish, meso- and metatibia whitish.

Head 1.08–1.28 times as broad as mesoscutum. POL 1.26–1.74 times OOL. Eyes 1.36–1.49 times as high as broad, separated by 1.34–1.53 times their height. Malar space 0.52–0.61 times eye height. Head in frontal view 1.23–1.31 times as broad as high. Antenna with scape 0.74–0.84 times as long as eye height. Combined length of pedicel plus flagellum 0.75–0.87 times head breadth. First and second anellus strongly transverse, third transverse, about as long as second anellus.

Mesosoma 1.18–1.41 times as long as broad. Mesoscutum 1.62–1.97 times as broad as long. Hind margin of scutellum broadly rounded. Upper mesepimeron strongly narrowing below, reaching at most basal third of mesopleuron. Basal setal line complete. Costal cell with dorsal surface at most

with a few setae in distal half, lower surface with at least two setal lines running towards proximal part, costal setal line complete. Fore wing disc moderately pilose. Marginal vein 1.64–2.30 times as long as stigmal vein. Stigma subcircular, small. Propodeum 0.30–0.52 times as long as scutellum. Median carina strong, straight. Median area in anterior part evenly reticulate with inner corner of anterior plica with a moderately deep and finely reticulate depression along the anterior plicae. Nucha subglobose, triangularly produced and delimited anteriorly by a fine carina, mostly smooth.

Metasoma: Gaster 1.90–3.12 times as long as broad, 1.05–1.28 times as long as mesosoma, and 0.49–0.76 times as long as mesoscutum. Posterior margin of second gastral tergite weakly incised medially. Posterior margin of third gastral tergite almost straight.

Material examined. Beside the above mentioned holotype we examined the following specimens: *Paratypes*, IVORY COAST: 14 ♂, 71 ♀, Lamto (*J.-Y. Rasplus*) (BMNH, CBGP, NMBE) (for label data, see Rasplus, 1988).

Further material examined, not belonging to the type series. BURKINA FASO: 1 ♀, Hauts-Bassins Region, Farakoba, ex "*Rezotrachelus vorium*" (? = *Piezotrachelus varius* Wagner, 1908) on *Vigna sinensis* Endl. ex Hassk., 2.iv.1967 (MHNG); KENYA: 1 ♀, Kwale County, Mombasa, Diani Beach, vii.1951 (*N.L.H. Krauss*) (BMNH); MALI: 1 ♂, 2 ♀, ex host on *Vigna* sp., (*R. Dugast*) (BMNH); NIGERIA: 4 ♂, 4 ♀, Kaduna State, Zaria, Samaru, ex host on *Vigna unguiculata*, xii.1962, associated with *Piezotrachelus varium* (= *Piezotrachelus varius*) (BMNH); 2 ♂, Kaduna State, Zaria, ex host on *Vigna unguiculata*, iv.1963 (BMNH); 7 ♂, 7 ♀, Kano Province, Kano, ex host on *Vigna unguiculata*, x.-xi.1961 (*P.F. Prevett*) (BMNH); 1 ♀, Oyo State, Ibadan, vi.1976 (*B.R. Critchley*) (BMNH); SENEGAL: 1 ♀, Diourbel Region, Bambey, 1941 (*J. Risbec*) (BMNH); 1 ♀, Diourbel Region, Bambey, (*J. Risbec*) (BMNH); 1 ♀, Diourbel Region, Bambey, 2.i.1942 (*J. Risbec*) (BMNH); SUDAN: 1 ♀, Khartoum, Shambat, ex host on *Abutilon* sp., 1.ii.1930 (*H.W. Bedford*) (BMNH); TOGO: 3 ♂, 5 ♀, Plateaux, Anié, Kolokope, xi.1990 (CBGP); YEMEN: 1 ♀, Ibb, Jebel Sumara, 2.i.1938 (*H. Scott* and *E.B. Britton*) (BMNH); 1 ♀, Jebel Sabir, 19.xii.1937 (*H. Scott* and *E.B. Britton*) (BMNH); ZIMBABWE: 2 ♀, Harare, Harare, (*A. Watsham*) (BMNH); 1 ♀, Makumbi.

Biology. Reared from *Piezotrachelus* sp. (Coleoptera: Apionidae) in pods of *Vigna multinervis* Hutch. & Dalziel, *V. ambacensis* Welw. ex. Bak. and *V. unguiculata* L. Walp. (Rasplus, 1988).

Distribution. Mainly Afrotropical region, but also further North in Sudan and Yemen.

Remarks. Paratype “2079 Baur” was misidentified by Rasplus (1988) and actually belongs to *A. calandrae*.

Anisopteromalus calandrae (Howard)

Further material examined, not belonging to the type series. ALGERIA: 1 ♀, Annaba Province, ex *Lasioderma serricorne* (Fabricius, 1792), ix.1935 (BMNH);

AUSTRALIA (?): 1 ♀, ex host on *Triticum* sp., in London ex Sydney (? via ship) (BMNH); BELGIUM: 3 ♂, 3 ♀, Antwerp Province, Antwerp, ex *Callosobruchus maculatus* (Fabricius, 1775) on *Cicer arietinum* L., xi.1947 (*R. Mayné*) (MHNG); 1 ♀, Antwerp, "Cappellen" (= Kapellen), ex *Callosobruchus maculatus* (Fabricius, 1775) on *Cicer arietinum* L., x.1947 (*R. Mayné*) (MHNG); 2 ♀, Antwerp, Antwerp, ex *Callosobruchus maculatus* (Fabricius, 1775), ix.1947 (*R. Mayné*) (ETHZ); 2 ♀, Antwerp, Antwerp, ex host on *Zea mays* L., 13.v.1948 (*R. Mayné*), ex diverse coleopterans in maize (MHNG); 1 ♀, Antwerp, Loenhout, ex *Callosobruchus maculatus* (Fabricius, 1775), ix.1947 (*R. Mayné*) (ETHZ); 1 ♂, Namur, Gembloux, ex *Sitophilus granarius* (Linnaeus, 1758) or *Sitophilus oryzae* (Linnaeus, 1763) (MHNG); BRAZIL: 1 ♀, Santa Catarina, Nova Teutonia, 27°11'B. 52°23'L., 8.ii.1937 (*F. Plaumann*) (BMNH); 5 ♀, Santa Catarina, Nova Teutonia, 3.ii.1950-5.iii.1950 (*F. Plaumann*) (BMNH); CHINA: 1 ♀, Guangdong, Guangzhou, Mount Xiqiao, 29.v.1983 (*Z. Boucek*) (BMNH); 1 ♂, 1 ♀, Henan, Luohe, ix.1989 (*Y. Zhao*) (UCD); HONG KONG: 1 ♂, Kowloon, Tsim Sha Tsui, ex *Lasioderma serricorne* (Fabricius, 1792) (?), 20.vii.1993 (*C.S.K. Lau*) (BMNH); COSTA RICA: 1 ♀, ex host on *Zea mays* L., 1985, stored host plant (BMNH); CYPRUS: 1 ♀, Nicosia District, Nicosia, ex *Lasioderma serricorne* (Fabricius, 1792) on *Pimpinella anisum* L., x.1959 (BMNH); CZECH REPUBLIC: 1 ♀, Hradec Králové Region, Sobotka, 23.viii.1974 (*Samsinak*) (BMNH); DEMOCRATIC REPUBLIC OF THE CONGO: 1 ♀, Tshibinda, viii.1931 (*T.D.A. Cockerell*) (BMNH); ECUADOR: 1 ♀, Napo Province, Huahua Sumaco, 15.-22.xii.1989 (*M.* and *J. Wasbauer*, *H. Real*) (UCD); ENGLAND: 34 ♂, 75 ♀, Berkshire, Slough, 1975 (*J.B. Southgate*), cultured 2002 on *Callosobruchus chinensis* (Linnaeus, 1758), laboratory culture (NMBE); 13 ♂, 5 ♀, Berkshire, Slough, 1975 (*J.B. Southgate*), cultured 1982 (BMNH); 8 ♂, 9 ♀, Berkshire, Slough, 1975 (*J.B. Southgate*), cultured 20.-22.iv.1998 on *Callosobruchus* sp. (NMBE); 1 ♀, Greater London, London, Lambeth, ex *Lasioderma serricorne* (Fabricius, 1792), 9.x.1977, host in "Weetabix" (BMNH); FIJI: 2 ♀, Viti Levu, Lautoka, 16.iv.1920 (*R. Veitch*) (BMNH); 1 ♂, Viti Levu, Lautoka, 14.x.1920 (*R. Veitch*) (BMNH); 1 ♂, Viti Levu, Lautoka, 20.i.1920 (*W. Greenwood*) (BMNH); 1 ♂, Viti Levu, Lautoka, 17.iv.1920 (*W. Greenwood*) (BMNH); FRANCE: 1 ♂, 2 ♀, Provence-Alpes-Côte d'Azur, Toulon, ex *Sitophilus oryzae* (Linnaeus, 1763) (ETHZ); GERMANY: 6 ♂, 18 ♀, Bayern, Eggmühle, 2002, laboratory culture (NMBE); 1 ♀, Hamburg, Hamburg, ex *Araecerus fasciculatus* (De Geer, 1775) on *Coffea* sp., x.1950 *(H. Weidner*) (BMNH); 1 ♂, 2 ♀, Hamburg, Hamburg, ex *Araecerus fasciculatus* (De Geer, 1775) on *Coffea* sp., 1950 (*H. Weidner*) (MHNG); GHANA: 1 ♀, "Eastern Province" (= Eastern Region), "Aleuri" (?), ex *Sitophilus oryzae* (Linnaeus, 1763) on *Zea mays* L., i.1935 (*G.S. Cottérell*) (BMNH); GREECE: 6 ♂, 11 ♀, Attica, Athens, ex *Sitophilus zeamais* (Motschulsky, 1855), 2004, laboratory culture (NMBE); INDIA: 2 ♂, 2 ♀, Andhra Pradesh, Rajahmundry, Central Tobacco Research Institute, ex *Lasioderma serricorne* (Fabricius, 1792) (BMNH); 1 ♀, Assam, Jorhat, Agricultural University, ex *Callosobruchus analis* Fabricius, 1781 on *Cicer arietinum* L., 30.xi.1989, host plant in laboratory

(BMNH); 1 ♀, Gujarat, Palanpur, Sardarkrushinagar, ex host on *Triticum aestivum* L., 21.ix.1987, stored host plant (BMNH); 1 ♀, National Capital Territory, Delhi, IARI area, 15.xi.1979 (*Z. Boucek*) (BMNH); 1 ♀, Tamil Nadu, Coimbatore, ex *Pempheres* sp. on *Gossypium* sp., vii.-viii.1934 (*P.N. Krishna Ayyar*) (ETHZ); 1 ♀, Uttarakhand, Dehradun, ex *Ophiomyia lantanae* Froggatt, 1919, 13.v.1937 (*N.C. Chatterjee*) (BMNH); 1 ♀, ex *Stegobium paniceum* (Linnaeus, 1758), i.1966 (*M. Meerzainuddin*) (BMNH); INDONESIA (?): 2 ♂, ex host on *Cocos nucifera* L., i.1974, on copra pellet residues on ship in Gladstone dock, Liverpool (BMNH); IRAN: 2 ♂, 4 ♀, Alborz, Karaj, ex *Callosobruchus maculatus* (Fabricius, 1775) (*Chodjai*) (MHNG); 2 ♀, Alborz, Karaj, ex *Callosobruchus maculatus* (Fabricius, 1775) (*Chodjai*) (BMNH); 3 ♂, Alborz, Karaj, ex *Callosobruchus maculatus* (Fabricius, 1775), viii.1973 (*Chodjai*) (BMNH); 1 ♂, 2 ♀, Alborz, Karaj, ex *Sitophilus granarius* (Linnaeus, 1758) on *Triticum* sp. (MHNG); 1 ♂, 2 ♀, Bushehr Province, Bushehr, *ex Callosobruchus maculatus* (Fabricius, 1775) on *Phaseolus vulgaris* L. (MHNG); 9 ♂, 11 ♀, Tehran Province, Tehran, ex *Callosobruchus maculatus* (Fabricius, 1775) on *Pisum* sp., xi.2002 (*F. Kazemi*) (NMBE); ISRAEL: 1 ♀, ex "*Myelois ceratoniae*" (= *Ectomyelois ceratoniae* Zeller, 1839) (MHNG); ISRAEL/LEBANON (?): 2 ♀, Upper Galilee, ex host on *Olea europaea* L., ix.1958 (*Gavrielit*) (?) (BMNH); ITALY: 1 ♀, Emilia-Romagna, Mirandola, viii.1996 (*A. Cazzuoli*) (NMBE); JAPAN: 6 ♀, Chiba, Ichihara, Anegasaki, 19.viii.1999 (*H. Kang*) (UCD); KENYA: 2 ♀, Kilifi County, Mtwapa, ex host on *Cajanus cajan* (L.) Millsp., 18.xi.1972 (BMNH); LIBYA: 1 ♂, 1 ♀, Greater Tripoli, Tripoli, ex *Lasioderma serricorne* (Fabricius, 1792), 1955 (*H. Martin*) (MHNG); MALAYSIA: 1 ♂, 2 ♀, Kuala Lumpur Province, Kuala Lumpur, ex *Lasioderma serricorne* (Fabricius, 1792), *Callosobruchus maculatus* (Fabricius, 1775), 1975 (*Y. Koh Chen*), mixed with specimens of *A. quinarius* sp. n. on the same pin (BMNH); MALI: 1 ♀, Sikasso Province, Sikasso, x.1986 (*B. Sauphanor*), ex host on stock millet (NMBE); MAURITIUS: 4 ♀, ex *Corcyra cephalonica* (Stainton, 1866), 1942 (*R. Mamel*), in breeding rooms (BMNH); MEXICO: 2 ♀, Montecillo, ex *Callosobruchus maculatus* (Fabricius, 1775), x.1997 (*N. Barcenas Ortega*) (UCD); MOROCCO: 1 ♀, Rabat-Salé-Zemmour-Zaer, Rabat, 13.v.1963 (ETHZ); NIGERIA: 5 ♀, Oyo State, Ibadan, 25.-28.x.1913 (*W.A. Lamborn*) (BMNH); 4 ♀, Oyo State, Ibadan, (*W.A. Lamborn*) (BMNH); 3 ♂, Oyo State, Ibadan, 24.-26.x.1913 (*W.A. Lamborn*) (BMNH); PAKISTAN: 3 ♂, Sind, Karachi, ex *Callosobruchus maculatus* (Fabricius, 1775), 28.vi.1964 (*Azim*) (BMNH); 2 ♀, Sind, Karachi, ex *Callosobruchus maculatus* (Fabricius, 1775), 28.vi.1964 (BMNH); 2 ♀, Sind, Karachi, 7.iii.1974 (*Roomi*) (BMNH); PHILIPPINES: 2 ♂, Laguna, Los Banos, ex *Callosobruchus chinensis* (Linnaeus, 1758), 14.iv.1932 (*S.R. Capco*) (BMNH); 3 ♀, Laguna, Los Banos, 14.i.1939 (*L.B. Uichanco*) (BMNH); 1 ♀, Laguna, Los Banos, 2.iii.1933 (*L.B. Uichanco*) (BMNH); SENEGAL: 1 ♀, 1984, assoc. with *Sitotroga cerealella* (Olivier, 1789) and stored millet (BMNH); SOUTH KOREA: 1 ♀, Seoul National Capital Area, Seoul, 1987 (*Ryoo*), ex culture of *Lariophagus* sp. (BMNH); SPAIN: 10 ♂ 10 ♀, Barcelona, Caldes de Montbui, 2010 (*Belda*), ex culture of *Sitophilus oryzae*

(NMBE); SRI LANKA: 2 ♀, Central Province, Peradeniya, Gannoruwa, ex *Callosobruchus maculatus* (Fabricius, 1775), 11.iv.1989 (*G. Wijesekera*) (BMNH); 1 ♂, 2 ♀, Makandura/Gonawila (?), in legume seeds (bruchids) (BMNH); 1 ♀, x.1975, assoc. with *Callosobruchus maculatus* (BMNH); 1 ♀, Southern Province, Matara, University of Ruhuna, ex host on *Vigna radiata* (L.) R.Wilczek, 10.iii.1992 (*K. Senaratne*) (BMNH); SRI LANKA (?): 2 ♀, ex host on *Cocos nucifera* L., i.1974, on dessicated coconut on ship in Gladstone dock, Liverpool (BMNH); SWITZERLAND: 1 ♀, canton Basel, Basel, ex *Sitophilus granarius* (Linnaeus, 1758), 1.iv.1975 (*Renfer*) (BMNH); 8 ♂, 5 ♀, canton Zürich, Zürich, cultured i.1997 on *Callosobruchus chinensis* (Linnaeus, 1758), laboratory culture (ETHZ); SYRIA: 3 ♂, 11 ♀, Deir ezzor Province, Deir ezzor, Euphrat, ex *Rhyzopertha dominica* (Fabricius, 1792) on *Triticum* sp., viii.2001 (*R. Yakti*) (NMBE); TOGO: 2 ♂, 2 ♀, ex host on *Zea mays* L., 1990 (BMNH); TURKEY: 1 ♀, Izmir, Bornova, Kaskaloglu, ex *Lasioderma serricorne* (Fabricius, 1792) (NMBE); 3 ♀, Izmir, Bornova, ex *Callosobruchus maculatus* (Fabricius, 1775) (*Karman*) (MHNG); 1 ♂, Izmir, Bornova, ex *Lasioderma serricorne* (Fabricius, 1792) (MHNG); 1 ♂, Izmir, Söke, ex *Rhyzopertha dominica* (Fabricius, 1792) on *Triticum* sp., stored host plant (NMBE); 4 ♂, 14 ♀, Izmir, Söke, ex *Rhyzopertha dominica* (Fabricius, 1792) on *Triticum* sp., stored host plant (MHNG); 1 ♀, (*Kepsutlu*) (MHNG); UGANDA: 2 ♂, 3 ♀, Buikwe District, Ngogwe, ex host on *Zea mays* L., 16.i.1945 (*H.S. Darling*), stored host plant (BMNH); 1 ♂, Kampala District, Buundo, ex host on *Zea mays* L., 16.i.1945 (*H.S. Darling*), stored host plant (BMNH); 7 ♂, 10 ♀, Kampala District, Kampala, ex host on *Zea mays* L., 6.ii.1945 (*H.S. Darling*), stored host plant (BMNH); 11 ♀, Kampala District, Kyagwe, ex host on *Zea mays* L., 16.i.1945 (*H.S. Darling*), stored host plant (BMNH); 2 ♀, Kasese District, Kagando, ex host on *Zea mays* L., 16.i.1945 (*H.S. Darling*), stored host plant (BMNH); USA: 1 ♂, 3 ♀, California, Tulare, ex *Callosobruchus maculatus* (Fabricius, 1775) on *Vigna unguiculata* (L.) Walp., 10.x.1995 (UCD); 1 ♀, Georgia, Atlanta, ex *Sitophilus oryzae* (Linnaeus, 1763), 9.iv.1937 (*T. O'Neill*) (NHMV); 7 ♂, 27 ♀, Georgia, Savannah, ex *Sitophilus oryzae* (Linnaeus, 1763), ix.1978, cultured iii.2002 on *Lasioderma serricorne* (Fabricius, 1792), laboratory culture, same strain as neotype (ETHZ, MHNG, NHMV, NMBE, USNM, ZIN, ZMMU); 17 ♂, 32 ♀, Georgia, Savannah, ex *Sitophilus oryzae* (Linnaeus, 1763), ix.1978, cultured iii.2002 on *Sitophilus granarius* (Linnaeus, 1758), laboratory culture, same strain as neotype (ETHZ, MHNG, NHMV, NMBE, USNM, ZIN, ZMMU); 4 ♂, 40 ♀, South Carolina, Bamberg, ex *Sitophilus oryzae* (Linnaeus, 1763), ix.1992, cultured iii.2002 on *Sitophilus granarius* (Linnaeus, 1758), laboratory culture (NMBE); USA (?): 2 ♀, Pullman (?), Armstrong Station (?), ex host on *Hordeum vulgare* L., 11.xi.1957 (*D.W. Tuff*) (UCD); VENEZUELA: 2 ♂, 3 ♀, ex *Lasioderma serricorne* (Fabricius, 1792), x.1995 (*P. Cox*), reared in UK (BMNH); ZIMBABWE: 1 ♂, 2 ♀, Harare Province, Harare, (*A. Watsham*) (BMNH); 2 ♀, ex host on *Triticum* sp., ix.1916 (*W.W. Froggatt*), from bag of weevily wheat (BMNH); 6 ♀, ex host on *Triticum* sp., ix.1916, from bag of weevily wheat (BMNH); 3 ♂, 3 ♀, ex host on *Glycine*

max (L.) Merr., 25.i.1973,from soya meal residues; on ship intercepted Liverpool (BMNH); 1 ♀, ex host on *Triticum* sp. (BMNH).

Anisopteromalus caryedophagus Rasplus (Figs 6B, D, F)

Anisopteromalus caryedophagus Rasplus, 1988: 123–125: Holotype ♀ in MNHN, labeled "LAMTO – RCI 5°02W 6°13N [print] 5.3.1986 [hand]; ex gou. Piliostigma thonningii para. Caryedon serratus [print]; Anisopteromalus caryedophagus n. sp JY Rasplus det. 88; HOLOTYPE [print, red label]" (entire; glued on card rectangle); type locality: IVORY COAST: Lamto (examined by Baur).

Diagnosis, female. Head and mesosoma dark blue to blue-green, setae whitish, inconspicuous. Gena terete, not carinate near mouth margin (Fig. 6D). Flagellum at most slightly clavate, first funicular segment subconical, basally slightly broader than third anellus, provided with 1–2 rows of longitudinal sensilla. Scutellum projecting at level of anterior margin of dorsellum, in lateral view weakly curved (Fig. 6B). Fore wing with setae on wing disc dark. Speculum bare, widely open below. Anterior plica of propodeum short and strongly bent inwards, joining a strong costula, often with some longitudinal carinulae behind costula (Fig. 6F). Posterior margin of first gastral tergite only weakly curving backwards, not produced medially. Head breadth 1.35–1.55 times metatibia length and 1.52–1.69 times eye distance; head height 2.24–2.65 times eye breadth; eye height 1.06–1.22 times scutellum length; pedicel plus flagellum 1.50–1.74 times eye height; mesosoma length 8.69–10.71 times OOL; scutellum length 1.89–2.52 times stigmal vein; metatibia length 1.74–2.11 times marginal vein; gaster length 8.81–12.50 times OOL.

Description, female. Antenna with scape testaceous, pedicel testaceous, first and second anellus testaceous, third anellus testaceous (specimens from Brazzaville) or fuscous (specimens from Lamto), and rest of flagellum fuscous. Legs with tibiae testaceous, slightly infuscate in basal half.

Head 0.99–1.15 times as broad as mesoscutum. POL 1.76–2.29 times OOL. Eyes 1.47–1.67 times as high as broad, separated by 1.14–1.31 times their height. Malar space 0.43–0.55 times eye height. Head in frontal view 1.19–1.35 times as broad as high. Antenna with scape 0.71–0.85 times as long as eye height. Combined length of pedicel plus flagellum 0.77–0.87 times head breadth. First and second anellus strongly transverse, third conspicuous, about as long as the first two anelli.

Mesosoma 1.17–1.36 times as long as broad. Mesoscutum 1.72–2.13 times as broad as long. Hind margin of scutellum broadly rounded. Upper mesepimeron moderately narrowing below, almost reaching base of mesopleuron. Basal setal line complete. Costal cell with dorsal surface with a few setae in distal half, lower surface with at least two setal lines in proximal part, costal setal line complete. Fore wing disc moderately pilose. Marginal vein 1.50–

2.13 times as long as stigmal vein. Stigma subcircular, small. Propodeum 0.33–0.51 times as long as scutellum. Median carina irregular but distinct. Median area in anterior part evenly reticulate with inner corner of anterior plica with a rather deep and almost smooth depression along the anterior plicae. Nucha subglobose, often separated from rest of propodeum by distinct edge, mostly smooth.

Metasoma: Gaster 1.37–2.18 times as long as broad, 0.97–1.40 times as long as mesosoma, and 0.61–1.11 times as long as mesoscutum. Posterior margin of second and third gastral tergite incised medially.

Material examined. Beside the above mentioned holotype we examined the following specimens: *Paratypes*, IVORY COAST and REPUBLIC OF THE CONGO: 52 ♂, 223 ♀, (BMNH, CBGP, NMBE) (for label data, see Rasplus, 1988).

Biology. Reared in Ivory Coast from *Caryedon serratus* (Olivier) (Coleoptera: Chrysomelidae, Bruchinae) in pods of *Piliostigma thonningii* (Schumach.) Milne-Redh. and in pods of several *Cassia* sp. (Fabaceae: Caesalpinioideae); in Congo from *C. serratus* and *C. congensis* Decelle in *Piliostigma thonningii*, from *C. maculipes* Pic in *Cassia occidentalis* L. and from *Caryedon crampeli* Pic (Rasplus, 1988).

Distribution. Afrotropical region.

Remarks. The species can be recognized by the characters given in the key and diagnosis. It is quite similar to *A. quinarius* sp. n. Depending on locality, two forms are discernible according to the color of the third anellus (Brazzaville pale, Lamto fuscous) and ratio of gaster (Brazzaville 1.70–2.18; Lamto 1.40–1.90). While the difference in coloration of the anellus might simply represent some intraspecific variation, the difference in gaster proportions is probably due to alcohol treatment.

Anisopteromalus ceylonensis Sureshan (Fig. 6A)

Anisopteromalus ceylonensis Sureshan, 2010: 1123–1125: Holotype ♀ in UCD, labeled “SRI LANKA: Central Province, Kandy Dist. Vict. Randenigala Rantembe Sanct. Mahewele R., N07°12’ E080°57’ 25–29.VIII 1999 Malaise Trap M. & J. Wasbauer [print]; Anisopteromalus ♀ ceylonensis sp. n. Det. Sureshan. 2010 [hand]; Holotype [hand, red label]” (entire; glued on card point); type locality: SRI LANKA: Central Province, Vict. Randenigala Rantembe Sanct., Mahewele R. (examined by Baur).

Diagnosis, female. Head and mesosoma dark blue, setae whitish, conspicuous. Gena terete, not carinate near mouth margin. Flagellum at most slightly clavate, first funicular segment provided with 1–2 rows of longitudinal sensilla. Scutellum projecting at level of anterior margin of dorsellum, in lateral view strongly curved (Fig. 6A). Fore wing with setae on wing disc dark. Speculum bare, widely open below. Anterior plica of propodeum short and evenly curved, joining an indistinct costula. Posterior margin of first gastral tergite apparently curved backwards, not

produced medially (actually the base of gaster is distorted in the holotype, hence this character was difficult to assess). Head breadth 1.54 times metatibia length and 1.62 times eye distance; head height 2.02 times eye breadth; eye height 1.16 times scutellum length; pedicel plus flagellum 1.61 times eye height; mesosoma length 8.85 times OOL; scutellum length 2.15 times stigmal vein; metatibia length 1.56 times marginal vein; gaster length 14.23 times OOL.

Description, female. Antenna with scape testaceous, pedicel brown, flagellum brown. Legs with tibiae yellowish.

Head 1.13 times as broad as mesoscutum. POL 2.09 times OOL. Eyes 1.39 times as high as broad, separated by 1.19 times their height. Malar space 0.43 times eye height. Head in frontal view 1.33 times as broad as high. Antenna with scape 0.68 times as long as eye height. Combined length of pedicel plus flagellum 0.83 times head breadth. First and second anellus strongly transverse, third transverse, about as long as second anellus.

Mesosoma 1.2 times as long as broad. Mesoscutum 2.16 times as broad as long. Hind margin of scutellum broadly rounded. Upper mesepimeron strongly narrowing below, reaching at most basal third of mesopleuron. Basal setal line complete. Costal cell with dorsal surface with a single row of setae over the whole length, lower surface with a patch of setae in distal half and a single row of setae running to proximal part, costal setal line complete. Fore wing disc moderately pilose. Marginal vein 2.02 times as long as stigmal vein. Stigma subcircular to oval, medium-sized. Propodeum 0.42 times as long as scutellum. Median carina fine, straight. Median area in anterior part evenly reticulate with inner corner of anterior plica reticulate, about as strong as on median area. Nucha subglobose, not distinctly separated from rest of propodeum, alutaceous.

Metasoma: Gaster 2.57 times as long as broad, 1.61 times as long as mesosoma, and 0.75 times as long as mesoscutum. Posterior margin of second gastral tergite incised medially, third gastral tergite strongly incised.

Material examined. Beside the above mentioned holotype we examined the following specimens: *Paratypes*, 4 ♂, same data as holotype. We received a fifth male from UCD also labeled as paratype and with the same data, although Sureshan (2010: 1124) reported only 4 paratypes. It obviously belongs to a different genus and is thus excluded from the type series.

Biology. Unknown.

Distribution. ASIA: Sri Lanka (Sureshan, 2010).

Remarks. The species is similar to *A. caryedophagus* as already mentioned by Sureshan (2010). It can be easily recognized by the characters and ratios given in the key.

Anisopteromalus quinarius Gokhman & Baur sp. n.

Further material examined, not belonging to the type series. AUSTRALIA: 1 ♀, Queensland, Brisbane, 16.iv.1967 (BMNH); CHINA: 5 ♀, 9.ii.1989, assoc. with *Lasioderma serricorne* (UCD); EGYPT: 1 ♀, Gharbia Governorate, Tanta,

22.vii.1918 (BMNH); 1 ♀, New Valley Governorate, Kharga, Kharga Oasis, ex host on *Phoenix dactylifera* L., 22.v.1916 (BMNH); 1 ♂, New Valley Governorate, Kharga, Kharga Oasis, ex host on *Phoenix dactylifera* L., 10.viii.1916 (BMNH); 1 ♀, ex host on *Pluchea dioscoridis* (L.) DC., 1.x.1919 (BMNH); FRANCE: 1 ♀, Réunion Island, St. Denis, 1982 (*B. Vercambre*) (CBGP); GERMANY: 1 ♀, (*F. Zacher*) (BMNH); INDIA: 2 ♀, Karnataka, Vittal, ex *Areca* sp., 26.viii.1976, from stored areca nuts (BMNH); MALAYSIA: 3 ♂, 4 ♀, Kuala Lumpur Province, Kuala Lumpur, ex *Lasioderma serricorne* (Fabricius, 1792), *Callosobruchus maculatus* (Fabricius, 1775), 1975 (*Y. Koh Chen*), mixed with specimens of *A. calandrae* on the same pin (BMNH); MOZAMBIQUE: 1 ♂, 1 ♀, 1956, assoc. with *Calandra* sp. (BMNH); NIGERIA: 1 ♀, Rivers State, Port Harcourt, Cocoa Warehouse, 31.v.1961 (*J.A. Freeman*) (BMNH); RUSSIA: 11 ♂, 7 ♀, Moscow Province, Moscow, cultured 20.-22.iv.1998 on *Sitophilus granarius* (Linnaeus, 1758) (NMBE); SOLOMON ISLANDS: 1 ♂, 2 ♀, Guadalcanal, 1985 (*R. MacFarlane*) (BMNH); SPAIN: 3 ♂, 3 ♀, Valencia Province, Valencia, ex *Lasioderma serricorne* (Fabricius, 1792), 17.viii.1981 (BMNH); SRI LANKA: 1 ♀, Central Province, Peradeniya, Hantana Mt., 7°15´N 80°37´E,10.-14.viii.1999 (*M.* and *J. Wasbauer*) (UCD); SWEDEN: 5 ♂, Skåne County, Helsingborg, ex *Lasioderma* sp., 10.xi.1957 (*T.-E. Leiler*) (BMNH); 5 ♂, Skåne County, Helsingborg, ex *Lasioderma* sp., 25.xi.1957 (*T.-E. Leiler*) (BMNH); SWITZERLAND: 2 ♂, 3 ♀, canton Zürich, Zürich, vii.1994 (*K. Dorn*), in house (ETHZ); 1 ♀, without data (*V. Delucchi*) (ETHZ).

Taxa not belonging to *Anisopteromalus* or with doubtful status

Anisopteromalus camerunus (Risbec)

Aplastomorpha camerunus Risbec, 1956: 238–240: Syntypes 2 ♂, 1 ♀ in MNHN; type locality: CAMEROON: Pouss.

Biology. Ex larvae of the rice gall midge *Pachydiplosis* (now *Orseolia*) *oryzae* (Wood-Mason, 1889) (Diptera: Cecidomyiidae) (Risbec, 1956: 240).

Remarks. According to Gérard Delvare [pers. comm.], who recently examined the types, this species clearly belongs to the genus *Cyrtoptyx*.

Status. *Cyrtoptyx camerunus* (Risbec) **comb. n.**

Anisopteromalus glaber Szelényi

Anisopteromalus glaber Szelényi, 1981: 215–216. Holotype ♀ in Hungarian Natural History Museum, Budapest, Hungary; type locality: GERMANY: Hessen, Schlitz, Breitenbachs.

Biology. Unknown.

Remarks. Unfortunately, it was not possible to see the holotype. From Szelényi's description *A. glaber* clearly does not belong to *Anisopteromalus*

(character states of the latter in brackets): “clypeus... anterior margin straightly obtuse” [slightly emarginate]; his Figure E shows a strongly clavate flagellum with all funicular segments strongly transverse [flagellum much less clavate and proximal funicular segments at least as long as broad]; “pronotum... collar very short, smooth and shining, anterior margin angled but without distinct carina” [pronotum collar relatively long, at least about one fifth or one sixth as long as mesoscutum length, wholly reticulate and anteriorly rounded]; “propodeum... with clearly cut plicae...” [plicae not clearly delimited, forming a rounded edge]; “propodeum... with a loose tuft of tolerably long, white hairs...” [propodeum without tuft of white hairs]; “Gaster... almost circular” [gaster elongate]. In fact, the characters mentioned by Szelényi almost perfectly fit the generic definition of *Meraporus* (e.g., Graham, 1969; Bouček & Rasplus, 1991) for which reason we put *A. glaber* in this genus.

Status. *Meraporus glaber* (Szelényi) **comb. n.**

Anisopteromalus schwenkei (Roomi, Khan & Khan)

Pteromalus schwenkei Roomi, Khan & Khan, 1973: 396–399: Holotype ♀ in Pesticides Research Division, P.C.S.I.R. laboratories, Karachi (Roomi et al., 1973: 396); type locality: PAKISTAN: Tandojam.

Biology. Reared from *Callosobruchus chinensis* (Linnaeus) [sub *Bruchus chinensis* Linnaeus] (Coleoptera: Chrysomelidae) on mung seeds (*Phaseolus mungo* L., Fabaceae).

Remarks. Mani (1989: 576) transferred the species to *Anisopteromalus*. Unfortunately, the holotype could not be obtained for examination, despite our efforts to contact some colleagues in Karachi. According to Bouček (pers. comm.), who also couldn’t get access to the types but examined some voucher specimens apparently originating from the same series as the types (see also Bouček et al., 1979: 436), that material consisted of a mixture of *Dinarmus* and *Anisopteromalus* species. Here we would rather like to point to one statement in the original description that gives a fair indication to which genus the species might belong. Roomi et al. (1973: 396) stated that in the female holotype the first anellus is shorter than the second and third and gave a figure of the female antenna (p. 398, Fig. 2A) that shows relatively elongate anelli. If, as we assume, this figure is more or less correct then any of the *Anisopteromalus* species can be ruled out. The figured antenna rather fits some species of *Dinarmus* (e.g., compare the figures of antenna in Rasplus, 1989), for which reason we suggest to put *P. schwenkei* in this genus.

Status. *Dinarmus schwenkei* (Roomi, Khan & Khan) **comb. n.**

Aplastomorpha vandinei brasiliensis Domenichini

Aplastomorpha vandinei var. *brasiliensis* Domenichini, 1951: 119–121: Syntypes ♂♂ and ♀♀, depository unknown; type locality: BRAZIL.

Biology. Reared from *Callosobruchus maculatus* (Fabricius) (Coleoptera:

Chrysomelidae).

Remarks. Domenichini did not state the depository of the type material. It might be deposited in the Istituto di Entomologia e Patologia in Piacenza, Italia (Renato Regalin, pers. comm.), but our requests in 2010 and 2011 ended without any result.

Aplastomorpha vandinei brasiliensis was actually described as a variety but following article 45.6.4 of the ICZN it has to be treated as a subspecies. Noyes (2013) put this taxon among the synonyms of *A. calandrae*. We assume that he did so because the Universal Chalcidoidea Database does not recognize the subspecies category. However, the taxon has never been synonymized in a revisionary study and must be considered as valid. Only De Santis (1980: 231) made the obvious change in combination from *Aplastomorpha vandinei brasiliensis* to *Anisopteromalus calandrae brasiliensis*.

From Domenichini's (1951: 119–121) description *A. c. brasiliensis* appears to be quite close to *A. calandrae* as we define it here, though we cannot be sure without having seen the types or topo-typical material. Evidently it differs from any of our new species. First, *A. quinarius* sp. n. is distinguished by relatively larger eyes, that is, the ratio eye distance to eye height is 1.12–1.35, while it is about 1.68 according to measurements made using Domenichini's figure VIIIb on page 119 of the head in frontal view; second, *A. cornis* sp. n. has a relatively longer flagellum resulting in a ratio of pedicel plus flagellum to head breadth of 1.06–1.26, while, as measured from the habitus drawing of an entire specimen in dorsal view (figure IX on page 121), the ratio is only about 0.92 for *A. c. brasiliensis* (unfortunately, Domenichini did not indicate measurements in his description, except for the lengths of individual antennal segments and veins of the fore wing).

Status. *Anisopteromalus calandrae brasiliensis* (Domenichini) **stat. rev.**, valid subspecies.

Neocatolaccus indicus Ayyar & Mani

Neocatolaccus indicus Ayyar & Mani, 1937: 126–127: Holotype ♀ with catalog no. 1572/H 3 in the collections of the Zoological Survey of India, Indian Museum, Calcutta; holotype dissected and mounted on 3 slides (Ayyar & Mani, 1937: 127); type locality: INDIA: Coimbatore (Bouček et al., 1979: 436) (not examined).

Biology. Reared from *Pempheres affinis* (Faust, 1898) (Coleoptera: Curculionidae).

Remarks. Noyes (2013) listed the species under *A. calandrae* following the synonymization of Bouček et al. (1979: 436). However, Mani (1989: 631) later put it under *Oxysychus sphenopterae* (Ferrière, 1931), as also stated by Noyes (2013, under *A. calandrae*). With regard to the original description of *N. indicus* we are inclined to follow Mani. For instance, Ayyar & Mani (1937: 126) stated that the first funicular segment is twice as long as the pedicel. This clearly rules

out any *Anisopteromalus* species treated here where the first funicular segment is always clearly shorter than twice the length of the pedicel.

Status. Junior synonym of *Oxysychus sphenopterae* (Ferrière, 1931) **syn. n.**

References

Ayyar, T.V.R. & Mani, M.S. (1937) On three chalcidoid parasites of cotton borer-beetles from South India. Records of the Indian Museum, **39**, 125–127.

Bouček, Z. & Rasplus, J.-Y. (1991) *Illustrated key to West-Palearctic genera of Pteromalidae (Hymenoptera: Chalcidoidea)*. Institut National de la Recherche Agronomique, Paris.

Bouček, Z., Subba Rao, B.R. & Farooqi, S.I. (1979) A preliminary review of Pteromalidae (Hymenoptera) of India and adjacent countries. *Oriental Insects*, **12**, 433–467.

De Santis, L. (1980) *Catálogo de los Himenópteros Brasileños de la serie parasítica incluyendo Bethyloidea.* Parana, Curitiba.

Domenichini, G. (1951) Contributo alla conoscenza del *Callosobruchus maculatus* F. e dei suoi parassiti. *Bolletino di Zoologia Agraria e Bachicoltura*, **17**, 101–122.

Graham, M.W.R.d.V. (1969) The Pteromalidae of North-Western Europe. *Bulletin of the British Museum (Natural History), Entomology, Supplement*, **16**, 1–908.

Mani, M.S. (1989) *The fauna of India and adjacent countries, Chalcidoidea (Hymenoptera. Part I). Agaontidae, Torymidae, Leucospidae, Chalcididae, Eurytomidae, Perilampidae, Eucharitidae, Cleonymidae, Miscogasteridae, Pteromalidae, Eupelmidae and Encyrtidae.* Zoological Survey of India, Calcutta.

Noyes, J.S. (2013) Universal Chalcidoidea Database. World Wide Web electronic publication. http://www.nhm.ac.uk/chalcidoids (accessed January 10th, 2013).

Rasplus, J.-Y. (1988) Description de deux nouvelles espèces du genre *Anisopteromalus* Ruschka. Clé des espèces Afrotropical [Hym. Pteromalidae]. *Bulletin de la Société Entomologique de France*, **93**, 119–127.

Rasplus, J.-Y. (1989) Révision des espèces afrotropicales du genre *Dinarmus* Thomson (Hymenoptera: Pteromalidae). *Annales de la Société Entomologique de France* (N. S.), **25**, 135–162.

Risbec, J. (1956) Les parasites des insectes borers du riz au Cameroun. *Agronomie Tropicale, Nogent-sur-Marne*, **11**, 234–247.

Roomi, M.W., Khan, Z.I. & Khan, S.A. (1973) *Pteromalus schwenkei* (Hymenoptera, Pteromalidae) a new species as a primary parasite of the bean-weevil, *Bruchus chinensis* L., from Pakistan. *Zeitschrift für*

Angewandte Entomologie, **72**, 395–400.

Sureshan, P.M. (2010) A new species of *Anisopteromalus* Ruschka (Hymenoptera: Chalcidoidea: Pteromalidae) from Sri Lanka. *Journal of Threatened Taxa*, **2**, 1144–1146.

Szelényi, G. (1981) Some new palaearctic torymid and pteromalid species (Hymenoptera: Chalcidoidea). *Acta Zoologica Academiae Scientiarum Hungaricae*, **27**, 211–216.
